# Genome of the facultative scuticociliatosis pathogen *Pseudocohnilembus persalinus* provides insight into its virulence through horizontal gene transfer

**DOI:** 10.1038/srep15470

**Published:** 2015-10-21

**Authors:** Jie Xiong, Guangying Wang, Jun Cheng, Miao Tian, Xuming Pan, Alan Warren, Chuanqi Jiang, Dongxia Yuan, Wei Miao

**Affiliations:** 1 Key Laboratory of Aquatic Biodiversity and Conservation, Institute of Hydrobiology, Chinese Academy of Sciences, Wuhan 430072, China; 2 University of Chinese Academy of Sciences, Beijing 100049, China; 3 Guangdong Ocean University, Zhanjiang 524088, China; 4 Lab of Protozoology, Department of Biology, College of Life Science and Technology, Harbin Normal University, Harbin 150025, China; 5 Department of Life Sciences, Natural History Museum, Cromwell Road, London SW7 5BD, United Kingdom

## Abstract

Certain ciliates of the subclass Scuticociliatia (scuticociliates) are facultative parasites of fishes in which they cause a suite of diseases collectively termed scuticociliatosis. Hitherto, comparatively little was known about genetics and genomics of scuticociliates or the mechanism of scuticociliatosis. In this study, a laboratory culture of the facultatively pathogenic scuticociliate *Pseudocohnilembus persalinus* was established and its genome sequenced, giving the first genome of a marine ciliate. Genome-wide horizontal gene transfer (HGT) analysis showed *P. persalinus* has acquired many unique prokaryote-derived genes that potentially contribute to the virulence of this organism, including cell adhesion, hemolysis and heme utilization genes. These findings give new insights into our understanding of the pathology of scuticociliates.

Scuticociliatosis, caused by certain scuticociliates^1^, is one of the most important parasitological problems in marine aquaculture worldwide^2^. In recent years there have been many reports of fatal outbreaks of infection by several scuticociliates species including *Pseudocohnilembus persalinus, Uronema marinum*, and *Philasterides dicentrarchi* in east Asia (Korea, Japan, China), Europe (Spain, Portugal) and other regions of the world, which have led to serious economic losses because of high mortalities of many fish species, in particular olive flounder and turbot^1^,^3^,^4^,^5^,^6^. Most studies of scuticociliatosis have focused on species identification, histopathology and immunology with little attention paid to molecular mechanisms of pathogenicity, mainly due to the lack of basic research on topics such as the life cycle, genetics and genome. Unlike the hymenostome ciliate *Ichthyophthirius multifiliis*, an obligate parasite of freshwater fish with a typical parasitic life cycle and distinct polymorphism^7^,^8^, scuticociliates are generally free-living in limnetic and marine ecosystems, feeding on other microorganisms such as bacteria, microalgae, protozoa etc^9^. Under certain circumstances, however, these ciliates may become opportunistic histophagous parasites, actively feeding on cells and tissue residues of a host organism, living and reproducing within the host tissues without observable morphological change.

Scuticociliates belong to the Scuticociliatia, one of six subclasses of the class Oligohymenophorea, the others being Peniculia, Hymenostomatia, Astomatia, Apostomatia, and Peritrichia^10^. Some oligohymenophoreans are the best-known of all ciliates and are commonly used as model laboratory organisms. These include the hymenostome species *Tetrahymena thermophila*^11^,^12^ and *I. multifiliis* and the peniculian species *Paramecium tetraurelia*^13^,^14^. Genomes of these ciliates have been sequenced and comparative genomics analyses have provided a comprehensive understanding of the unique features of these free-living (*T. thermophila* and *P. tetraurelia*) and typical parasitic (*I. multifiliis*) ciliates. Thus, it is expected that the genomes of parasitic scuticociliates will provide new insights and better understanding of their mechanisms of pathogenicity.

The typical *Pseudocohnilembus* species, *P. persalinus* has been reported previously as a free-living marine ciliate^15^,^16^,^17^. Since Kim etc. (2004) isolated *P. persalinus* from diseased olive flounder in Korea, it has become recognized as an important facultative parasite causing scuticociliatosis in commercially important fishes such as rainbow trout^18^ and olive flounder^19^. *Pseudocohnilembus persalinus* can be cultured in the laboratory as a free-living form by feeding with bacteria, which enables sufficient DNA/RNA to be collected for genome/transcriptome sequencing. Here, we report the genome of *P. persalinus*, the first to be sequenced among scuticociliates and, following comparative genomic analyses with its close relatives, investigate the possibility that the acquisition of its virulence may be via horizontal gene transfer (HGT) from bacteria.

## Results and Discussion

### Facultative parasitism of *P. persalinus* - a genomic view

**Figure 1:**
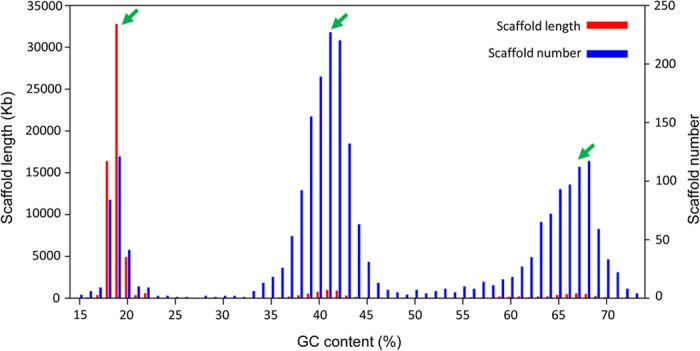
GC content distribution of preliminary genome assembly. Red, scaffold length versus GC content; blue, scaffold numbers versus different GC content. Green arrows, GC peaks by scaffold length.

Like most other ciliates, *P. persalinus* has two types of nucleus, a macronucleus (MAC) and a micronucleus (MIC), the former controlling the physiological and biochemical functions of the cell and the latter as a germ-line reserve. The first ciliate genome to be sequenced was the MAC genome of *T. thermophila*^12^ and this was achieved by first physically separating the MAC from the MIC. However, such a method has not been established for *P. persalinus*. In most ciliates the MIC is either haploid or diploid whereas the MAC is polyploid. In *T. thermophila*, for example, the MAC has an average ploidy up to 45C^11^, and in *I. multifiliis*, the MAC has an estimated ploidy up to 12,000C^20^. Therefore, we anticipated that the MAC of *P. persalinus* also has a high ploidy level and utilized this natural enrichment of MAC in order to perform MAC genome sequencing as had previously been achieved in *I. multifiliis*^20^. By employing this simple sequencing strategy, a preliminary assembly of about 63.6 Megabase (Mb) sequences with 2403 scaffolds was constructed. The GC content distribution of the preliminary assembly showed that there are three peaks of 19%, 41% and 67% (Fig. 1, red), indicating the presence of contaminants. Compared to the second and third peaks, the first peak contained a majority of sequences (sequence length, about 87%, Fig. 1, red) with a small population of scaffolds with low GC content (scaffold number, Fig. 1, blue). Published oligohymenophorean ciliate genomes usually show a low GC content (22% in *T. thermophila*, 15% in *I. multifiliis* and 28% in *P. tetraurelia*, Table 1), therefore sequences represented by the first peak probably derive from *P. persalinus*. In order to verify this, BLASTX searches were performed for all preliminary assembled sequences against the NCBI non-redundant protein sequences database. The results support the suggestion that sequences in the first peak belong to *P. persalinus*, whereas sequences in the second and the third peaks probably derive from bacterial sources. In order to exclude bacterial sequences, two steps were applied: 1) based on their distribution like in *I. multifiliis*^20^, filtering all scaffolds with GC content higher than 25%; 2) filtering those scaffolds with more than 50% BLASTX hits in the remaining sequences after first step, these most likely being of prokaryote origin. In practice, almost all the filtered scaffolds were removed in the first step, with only two additional short scaffolds (1.5 and 1.6 Kb, respectively) being removed at the second step. This suggests that the GC content is a good index for discriminating ciliate from bacterial sequences. Finally, about 55.5 Mb *P. persalinus* genome sequences with 288 scaffolds were obtained (Table 1). The N50 of the *P. persalinus* genome is about 368 Kb which is comparable to the genome assemblies of *T. thermophila* (521 Kb) and *P. tetraurelia* (413 Kb) (Table 1).

The genome of *P. persalinus* provides evidence of parasitism: 1) its genome size is similar to another ciliate fish pathogen, *I. multifiliis* (47.8 Mb), and is significantly smaller than those of free-living ciliates such as *T. thermophila* (103 Mb) and *P. tetraurelia* (72.1 Mb) (Table 1); 2) the *P. persalinus* genome harbors 13,186 predicted protein coding genes, which is about two-fold less than the free-living *T. thermophila* (Table 1); 3) protein domain analysis showed very similar domain composition between *P. persalinus* and *I. multifiliis*, not only in the types but also the number (Figure S1); 4) *Pseudocohnilembus persalinus* and *I. multifiliis* have a similar fraction of parasitic lifestyle-relevant gene families and a relatively high percentage of proteases compared to free-living ciliates (Table S1). Compared to *I. multifiliis, P. persalinus* has more proteases especially in the cysteine and serine classes (Table S1) which may be key to various functions of a parasitic lifestyle including immunoevasion, excystment/encystment, ex-sheathing, and cell and tissue invasion^21^,^22^. Besides the proteases, 106 *P. persalinus-*specific transporters (Table S2) were found when compared to *T. thermophila*, and these transporters were significantly enriched in the sodium ion, zinc ion, calcium ion, ammonium and phosphate transmembrane transport systems (Figure S2). Some of these transport systems may play important roles in unique aspects of parasite biology. Calcium, for example, is an important factor in the invasion of erythrocytes^9^, and has been shown to help the secretion of parasite proteins during the invasion process of *Toxoplasma gondii*^23^,^24^. Thus, calcium transporters may be involved in the invasion process in *P. persalinus*. Furthermore, we compared the gene compositions of *P. persalinus* to the well-known parasitic ciliate *I. multifiliis* and the free-living *T. thermophila.* The results showed that very few unique orthologs (74) shared in *I. multifiliis* and *P. persalinus* (two parasites) are absent in *T. thermophila* (Figure S3), suggesting *P. persalinus* has a distinct mode of parasitism compared to *I. multifiliis*.

Scuticociliates infect aquatic organisms opportunistically and occurrences of scuticociliatosis seem to be influenced by environmental conditions, such as temperature and salinity, and weakening of the host due to bacterial infection^25^. The processes and mechanisms of the transition from free-living to parasitic lifestyle are unknown. By sequencing the genome of *P. persalinus*, this study revealed a number of features associated with parasitism.

### Horizontal gene transfer (HGT) genes may play an important role in the virulence of *P. persalinus*

**Figure 2:**
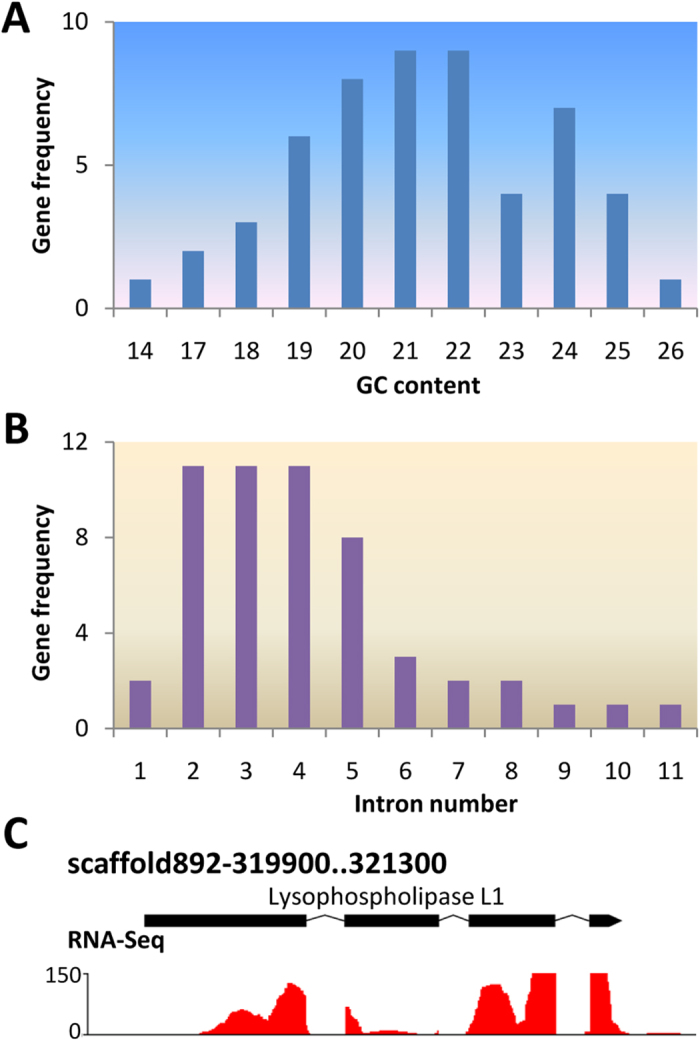
GC content and intron number distribution of the 54 HGT genes. (**A**) the GC content distribution, similar to the GC content distribution of the assembled scaffolds; (**B**) the distribution of the predicted intron numbers, only two genes lack introns. (**C**) a RNA-Seq supported intron-containing HGT gene (PPERSA_00047700). These results suggest that the 54 HGTs belong to the *P. persalinus* genome rather than to bacterial contaminants.

HGT, the transfer of genetic material between species^26^, was discovered half of a century ago^27^, but it is the current wealth of genomic sequence data that is revealing its real impact on evolution. Genes acquired by HGT can sometimes be associated with important evolutionary adaptations, including parasitism and pathogenicity^28^. The first evidence for the role of HGT in the acquisition of virulence determinants was between pneumococci in infected mice^29^. Subsequently, a number of studies have reported horizontal transfer of virulence genes (i) between prokaryotic pathogens, (ii) from prokaryotic to eukaryotic pathogens, and (iii) between eukaryotic pathogens^28^,^30^,^31^,^32^,^33^,^34^,^35^. In the obligate ciliate parasite *I. multifiliis*, which harbors an endosymbiotic bacterium, relatively few (10) HGT genes were predicted^20^. Using a phylogenetic approach (see Materials and Methods section), 74, 5, and 54 putative HGT genes were identified in *T. thermophila, I. multifiliis* and *P. persalinus*, respectively. The 54 putative HGT genes in *P. persalinus* were dispersed among 42 different assembled scaffolds (Table 2 and Supplementary Information), and had similar GC contents to the rest of the genes (Fig. 2A). PCR analysis of DNA also verified that these genes are present in the ciliate genome (Figure S4). In addition, 52 of these putative HGT genes were predicted to contain introns (Fig. 2B) which is a main feature of eukaryotic genes. Analysis of transcriptome (RNA-Seq) data reveals that 85% (44) of the putative HGT genes contain at least one intron (Fig. 2C). It is unlikely that the acquisition of introns by these HGT genes was for generating a more complicated proteome through alternative splicing because no alternative splicing was found in the RNA-Seq data. It is possible that the intron gains were the result of adaptation of the transferred gene to its new host cell machinery^36^. The presence of introns strongly suggest the origin of the putative HGT genes is the *P. persalinus* genome itself rather than bacterial contaminants, and that the HGT events occurred long ago in evolutionary time.

The number of HGT genes in *P. persalinus* is similar to that in the free-living species *T. thermophila*, and far higher than the obligate parasite *I. multifiliis.* Therefore, the HGT genes in *P. persalinus* and *T. thermophila* were compared. In *T. thermophila*, 15 HGT genes are homologs of chemotaxis proteins (Table S3) which are related to the movement of an organism in response to a chemical stimulus such as the presence of food^37^. Fourteen genes are tetratricopeptide (TPR) repeat family homologs (Table S3) which have a variety of functions. Six are protein kinases (Table S3), the kinase families being extensively expanded in *Tetrahymena* compared to other organisms^12^. In *P. persalinus*, a gene ontology enrichment analysis suggested that the HGT genes are significantly enriched in functions such as oxidoreductase activity and iron ion binding, which clearly differ from the HGT genes in *T. thermophila* (Figure S5). Therefore, we carefully checked the functional annotations of HGT genes in *P. persalinus* and found a number of HGT genes (approximately 20%) may play important role in its virulence.

#### Cell adhesion proteins

**Figure 3:**
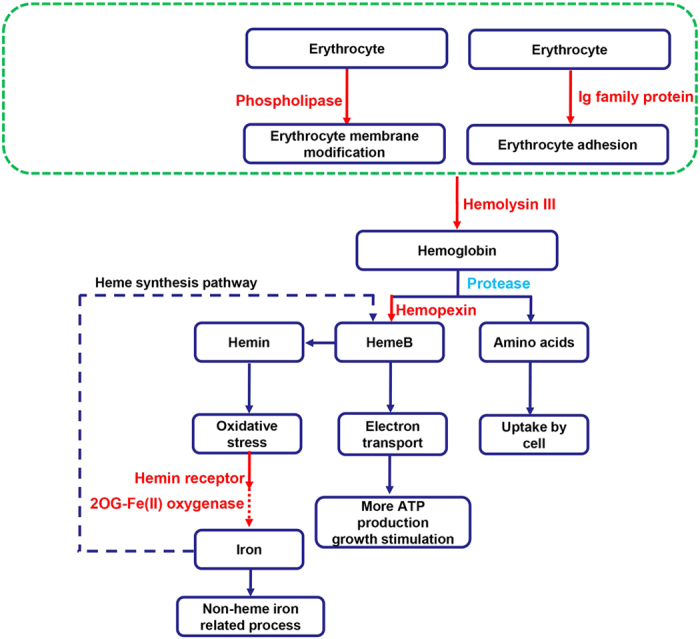
Potential contributions of HGT genes in virulence of *P. persalinus*. Red, HGT genes and their putative contribution to virulence; solid red arrow, processes and genes involved, supported by published studies. Red dashed arrow, potential function of HGT genes according to protein domain and gene regulatory information. Green dashed box, biological process in which the two Ig family proteins may be involved, although it is not known whether this process is necessary to the hemolysis pathway.

Two of the Ig family of proteins were found in the HGT genes of *P. persalinus*. Domain analysis of these genes showed that both contain cadherin-like domains (Figure S6). Cadherins are a family of transmembrane proteins that play important roles in cell adhesion, forming adherens junctions to bind together cells within tissues. They are dependent on calcium ions (Ca2+) to function hence their name. Cadherins have been shown as important adhesins and invasins of pathogenic bacteria^38^. Comparing the transporters between the facultatively parasitic *P. persalinus* and the free-living *T. thermophila* showed that the *P. persalinus-*specific transporter is significantly enriched in calcium ion transport (Figure S2 and Table S2). One of the two Ig proteins HGT genes in *P. persalinus* contain a He_pig domain (PF05345) which contains a conserved core region of about 90 residue repeats found in several haemagglutinins and other cell-surface proteins (http://www.ncbi.nlm.nih.gov/Structure/cdd/cddsrv.cgi?uid=pfam05345), indicating that this gene may contribute to cell (e.g. erythrocyte cell) adhesion (Fig. 3).

#### Hemolysis related proteins

It is well known that many bacterial pathogens induce hemolysis of host erythrocytes^39^. These bacteria could produce proteins, usually called hemolysin, that cause lysis of erythrocytes by destroying their cell membrane. Many proteins have been identified as hemolysins including phospholipase^40^ and hemolysin III^41^. Interestingly, three HGT phospholipase-related genes and a hemolysin III homolog were found in the *P. persalinus* genome (Table S2).

Bacterial phospholipases are a large group of enzymes that have a wide range of effects on host cells from minor alterations of cell membrane composition to increased vascular permeability and lethality, even at low concentrations^42^. In the scuticociliate *Uronema marinum*, it has been suggested that phospholipase C could acts as virulence factor that serves to actively disrupt host defense mechanisms^43^. Among the three phospholipases in *P. persalinus*, two (PPERSA_00098980 and PPERSA_00002080) were identified as phosphatidylinositol-specific phospholipase C (PI-PLC) and the other one (PPERSA_00047700) was identified as lysophospholipase (Table S2). Phospholipase has been reported to function in erythrocyte membrane modification and hemolysis, for example the PI-PLC, which could release acetylcholinesterase linked to phosphatidylinositol^44^,^45^,^46^,^47^. Thus, phospholipases may help *P. persalinus* utilize the host erythrocytes by disrupting their cell membrane (Fig. 3).

A hemolysin III family protein (Table 2, PPERSA_00035610) was identified among the HGT genes of *P. persalinus*. This gene is located in an assembled scaffold with 1.2 Mb in length and contains a RNA-Seq data-supported intron (Figure S7A). The coded protein contains a HlyIII domain (PF03006) and six transmembrane helices (Figure S7B). It has been shown that hemolysin III produced by the bacteria *Bacillus cereus* and *Vibrio vulnificus* is capable of hemolytic activity^48^. The *P. persalinus* hemolysin III gene closely resembles its *B. cereus* homolog (Figure S7C), especially in the functional domain region, suggesting that the protein for which this gene codes may play a role in the lysis of host erythrocytes (Fig. 3).

#### Heme related proteins

Hemolysis is the rupturing of the erythrocyte cell membrane and the release of its cytoplasm into surrounding tissue. By this process pathogens can acquire the erythrocyte cell contents and utilize it for their own metabolism. Erythrocyte cytoplasm is rich in hemoglobin which includes the iron-containing heme whose function is to bind and transport oxygen^49^. Hemoglobin can be digested by a series of protease enzymes, releasing its amino acids and heme. Amino acids can be directly intercepted by the pathogen, whereas free heme generates oxidative stress known to damage cells if not utilized or transformed^50^. In malaria, merozoites of *Plasmodium* invade erythrocytes, ingest host hemoglobin enclosing it in an acidic food vacuole^51^ and digest it using proteases^52^. The released heme is then incorporated into haemozoin^53^. Due to its abundance in the host, heme is a valuable source of iron for invading micro-organisms during hemolysis, and makes the host dramatically more susceptible to infections and their complications. *Pseudocohnilembus persalinus* has two hemopexin repeat protein homologs (PPERSA_00117390 and PPERSA_00079580) that appear to be of bacterial origin acquired by HGT (Table 2). These proteins are reported to have a high binding affinity for heme and are probably heme carriers^50^. The existence of these two HGT genes suggests that *P. persalinus* can uptake and utilize host iron in a similar way to bacteria (Fig. 3).

For many ciliates, e.g. *Tetrahymena*, the inclusion of inorganic iron salts in a culture medium produces a dramatic acceleration of growth and marked alterations in metabolism^54^. Iron supplementation could be used in heme synthesis and lead to an increased concentration or activity of certain heme enzymes, particularly in the electron transport chain which plays an important role in ATP synthesis thus stimulating cell growth^55^. In *P. persalinus*, the same heme de novo synthesis pathway was found as in *Tetrahymena* (Figure S8), suggesting that *P. persalinus* may also synthesize heme from iron in order to enhance growth. Therefore, it is reasonable to speculate that direct uptake of heme from the host could stimulate the reproduction of *P. persalinus* during infection (Fig. 3).

Hemin is the Fe(III) oxidation product of heme. An excess of hemin can interact with the cell membrane resulting in formation of reactive oxygen species (oxidative stress) and causing cellular injury^56^. For the host, the generation of hemin is a double-edged sword since it not only lyses pathogens^57^, but also induces hemolysis of erythrocytes^58^. Bacteria such as Y*ersinia enterocolitica* have evolved hemin utilization systems that enable them to acquire iron by intercepting hemin using hemin receptor proteins^59^. A hemin receptor gene of bacterial origin (PPERSA_00031570) has been acquired by *P. persalinus* (Table 2), indicating that the ciliate has the ability to use hemin. In addition to being a source of iron, the binding of free hemin by the hemin receptor may help to reduce the oxidative stress for the ciliate.

Heme, or its Fe(III) oxidation product hemin, is catalytically broken down by heme oxygenase to carbon monoxide, bilirubin and iron. The iron can then be used by non-heme iron enzymes or participate the de novo heme synthesis pathway. However, no homolog of heme oxygenase was found in *P. persalinus,* suggesting the presence of an alternative hemin utilization system. In the bacterium *Ralstonia metallidurans* CH34, for example, some hemin-related proteins share a transcription factor binding site (potential operon) with 2OG-Fe(II) oxygenase (http://regprecise.lbl.gov/RegPrecise/regulon.jsp?regulon_id=9937), indicating that 2OG-Fe(II) oxygenase may be involved in the hemin utilization process, although its function was not determined. In *P. persalinus*, two 2OG-Fe(II) oxygenase (PPERSA_00130810 and PPERSA_00076120) were found as HGT genes (Table 2), raising the possibility that 2OG-Fe(II) oxygenase could function as heme oxygenase, cleaving the hemin ring to release the iron (Fig. 3). Thus, it appears that *P. persalinus* acquired by HGT almost the whole pathway for hemolysis and the utilization of heme.

One of the most salient clinical manifestations of scuticociliatosis is haemorrhagic lesions^60^. Histopathological observations typically show many scuticociliates in the blood vessels, gills, fins, skin muscle, brain and lamina propria of the digestive tract, accompanied by necrosis and haemorrhages^61^. Like *Uronema marinum* which destroys host tissue by proteases^62^, *P. persalinus* may also utilize proteases to break the skin–blood barrier and gain entry to internal organs. Virulence HGT genes may thus contribute to the subsequent destruction of red blood cells and the acquisition of host-derived nutrients and energy for ciliate cell proliferation.

It has been reported that scuticociliatosis often accompanies bacterial disease (e.g. vibriosis), and the increased bacterial load probably helps ciliates to thrive and proliferate during the initial phase of infection^60^. Therefore it can be speculated that the synergistic invasion by pathogenic bacteria and scuticociliates, and the presence of both in a shared environment, may provide the opportunity for the transfer of genetic materials from the former to the latter. Alternatively, many ciliates harbor bacterial symbionts^63^ which may also provide the conditions for HGT. Evidence for the presence of endosymbiotic bacteria in *P. persalinus* comes from the preliminary genome assembly, the second and third peaks with 41% and 67% GC (Fig. 1) representing two bacterial species. The homology search results showed these could be species closely related to *Pseudoalteromonas* and *Halomonas*, respectively. They are very likely bacterial symbionts harbored in *P. persalinus* because sequences of *Escherichia coli* DH5 alpha, the only food organism supplied to *P. persalinus* cultured in laboratory, were not found. The 54 HGT genes in *P. persalinus* were not, however, included in these two peaks. Although homology searches showed that these 54 HGT genes do not have an enriched bacterial source, many of the best homologs occur in two large bacterial classes: Actinobacteria and Gammaproteobacteria (Table 2). Therefore, it is more likely that the HGT genes in *P. persalinus* have multiple independent origins.

Based on infection experiments, some researchers have concluded that the scuticociliates *P. persalinus, P. hargisi* and *U. marinum* are not primary pathogens of oysters, rather they are non-pathogenic, free-living, bacteriophagous and/or saprophagous ciliates that opportunistically attached to lesions on the host that are originally produced by bacterial infection or some other cause^61^. However, the identification here of virulence HGT genes in *P. persalinus* provides evidence that there is a molecular basis for its pathogenicity. Recent attempts to develop vaccines targeting antigens such as those responsible for host cell immobilization, proton-translocating inorganic pyrophosphatases, cathepsin L-like cysteine protease, etc. have met with limited success because of different levels of virulence and serotype-specific protection among species/strains of pathogens^64^,^65^,^66^,^67^. It is anticipated that the virulence HGT genes identified here will help us to gain a better understanding of the pathology of scuticociliatosis and provide potential antigen candidates for the development of anti-scuticociliatosis vaccines.

## Conclusions

In summary, we report the genome of scuticociliate *Pseudocohnilembus persalinus*, the first marine ciliate genome as far as we know. The genome of *P. persalinus* genome is 55.5 Mb, i.e., about half the size of the model free-living ciliate *Tetrahymena thermiphila*. The *P. persalinus* genome harbors many prokaryote-derived horizontally transferred genes; function analysis showed that many of these HGT genes are potential virulence factors. These findings help to increase our knowledge and understanding of the mechanism of the common fish disease, scuticociliatosis.

## Methods

### *Pseudocohnilembus persalinus* culture, total DNA and total RNA extraction

*Pseudocohnilembus persalinus* was isolated from water in a shrimp-farming pond (36°08′N,120°43′E; water temperature 27 °C; salinity 20%; pH 7.5) in Qingdao, China^68^. The species was identified by its morphology, morphogenesis^68^ and 18S rDNA marker (Figure S9). In order to obtain sufficient DNA and RNA material for sequencing, *P. persalinus* cells were cultured in the laboratory using sterilized sea water with *Escherichia coli* DH5 alpha as a food source. Contamination by bacteria was prevented by treating the cell cultures with lysozyme (200 μg/ml for 2 hours at 28 °C ) before the DNA and RNA extraction. The total DNA was extracted using the method described for *Tetrahymena*^69^, and the total RNA was extracted using the RNeasy Protect Cell Mini Kit (Qiagen, Valencia, CA) according to protocol in TetraFGD^70^.

### *Pseudocohnilembus persalinus* genome and transcriptome sequencing

The *P. persalinus* genome was sequenced using the Illumina platform. Paired-end (about 190 bp insert size) and mate-pair (about 2 Kb insert size) libraries were constructed and sequenced using the standard protocol of Illumina (https://icom.illumina.com/). Briefly, genomic DNA was fragmented and fragments of appropriate size (see above) were selected. For mate-pair library construction, fragment ends were biotinylated and circularized, and the fragments were then enriched using biotin. Fragment ends were then repaired, A-tailed and ligated with sequencing adaptors. Adaptor-ligated fragments were PCR amplified using Phusion polymerase, denatured with sodium hydroxide and diluted in hybridization buffer. The prepared libraries were loaded onto the flowcell and sequenced.

For transcriptome sequencing, Poly-A mRNAs were isolated using Dynal magnetic beads (Invitrogen) and fragmented by heating to 94 °C. First strand cDNAs were synthesized with reverse transcriptase and random hexamer primers, and then the second strands were synthesized with DNA polymerase and random hexamer primers. Double strand cDNAs were end-repaired and a single adenosine moiety was added. Illumina adapters were ligated and gel-electrophoresis was used to select DNA fragments about 200 bp size. Libraries were PCR-amplified using Phusion polymerase. Cluster formation, primer hybridization and pair-end sequencing were performed using proprietary reagents according to manufacturer-recommended protocols (https://icom.illumina.com/).

### Genome assembly and bacterial contamination exclusion

The paired-end reads and mate-paired reads of the Illumina sequencing were assembled using SOAPdenovo software^71^, which uses the de Bruijn graph data structure to construct contigs. A series of K-mer values (from 33 to 79) were used to assemble the *P. persalinus* genome, and the assembly with the longest N50 length was chosen by deleting scaffolds shorter than 1 Kb. Bacterial contaminants were excluded in two stages, sample preparation (see above) and bioinformatics analysis. In the bioinformatics analysis stage, bacterial contaminants were first excluded using the GC content, any scaffolds with a GC content more than 25% being discarded. The remaining scaffolds were then BLAST searched against the NCBI non-redundant protein database; any scaffolds with more than 50% hits belonging to the prokaryotes were excluded. The remaining scaffolds were regarded as the *P. persalinus* genome assembly sequences.

### Gene prediction and annotation

Using the RNA-Seq data, transcripts were both de novo assembled using Trinity^72^, and reference-guided assembled using the Tophat^73^ and Cufflinks^74^ pipeline. A combination of de novo and reference-guided assembled transcripts were validated by aligning the putative transcripts onto the assembled genome using PASA^75^. A set of the so-called best models generated by PASA was used to train the gene prediction software Augustus^76^ and GlimmerHMM^77^. The training parameters were then used by the two programs to de novo predict the gene models. The Augustus software could accept the cDNA or protein evidence, therefore the assembled transcripts were also used as the cDNA evidence for Augustus. Finally, an integrated set of gene models was created using Evidence Modeler^78^ by merging all of the predicted gene models.

Homologs of *P. persalinus* genes were BLAST searched against the NCBI non-redundant protein database. The KEGG pathway information was annotated using the KAAS server (http://www.genome.jp/kegg/kaas/). Protein domain information was annotated using the Pfam database^79^. Gene ontology (GO) information was annotated using the Goanna server (http://agbase.msstate.edu/cgi-bin/tools/GOanna.cgi). For each gene set, GO enrichment analysis was also carried out using BinGO^80^. FDR correction was used to control the false positive rate. If a GO term in a test gene set showed a corrected p value less than 0.05 compared with the reference set (all the GO annotated genes), the GO term (function) was determined to be significantly overrepresented in the test gene set. To annotate the proteases, a batch BLAST (http://merops.sanger.ac.uk/cgi-bin/batch_blast) was performed against the MEROPS database using all predicted genes. Genes with BLAST hit E-values less than 1e-10 were regarded as proteases, and the best hit in the MEROPS database was used to assign the protease classes. To annotate the membrane transporters, all the genes were BLAST searched against the TCDB transporters database^81^, and the transmembrane helices were predicted using SCAMPI^82^, Toppred^83^ and TMHMM^84^. A gene was regarded as a membrane transporter if it had a BLAST hit in the TCDB transporters database with an E-value less than 1e-03 and showed at least one transmembrane helix in all three programs.

### Bacterial horizontal transferred genes identification

*Pseudocohnilembus persalinus* HGT genes were identified by two steps, similar to the strategy used in Ricard *et al.*^85^: Firstly, similarity searches were performed to screen the potential prokaryotic origin genes by using a BLASTP search against to the NCBI non-redundant database (Figure S10). In this step, the E-value 1E-05 was used as the cutoff, and if a *P. persalinus* gene had a best hit belonging to the prokaryotes, it was regarded as a candidate gene. Phylogenetic approaches are widely used to identify HGT genes^85^,^86^,^87^, so we also employed phylogenetic analyses to to further identify the *P. persalinus* HGT genes based on the screened candidates. All candidate genes retrieved from the first step were BLASTP searched (E-value: 1E-05) against both the prokaryote and eukaryote protein databases (generated from the Refseq data: ftp://ftp.ncbi.nlm.nih.gov/refseq/release/) in order to retrieve homologs in both eukaryotes and prokaryotes. For a protein in *P. persalinus*, if there were homologs (E-value: 1E-05) in both domains, the sequences were retrieved in order to construct phylogenetic trees. Two methods (programs) were used, namely FASTTREE^88^ and PHYML^89^. For FASTTREE, all the homologs with E-value less than 1E-05 were used to construct the tree; for PHYML, only the top ten homologs (if present) were used to construct the tree. Sequences alignments were performed using MUSCLE^90^. A gene clustered in the prokaryotic clade which had a eukaryotic outgroup was accepted as an HGT gene, a technique now widely used to identify HGT genes^85^,^86^,^87^,^91^. As shown in Figure S11, only gene in *P. persalinus* with this kind of tree topology was considered an HGT gene. If a *P. persalinus* gene only has homologs with E-values less than 1E-05 in prokaryotes, the phylogenetic analysis could not work. HGTs were determined if there were at least 5 prokaryotic hits and the E-value of the best hit in prokaryotes and eukaryotes differed by more than 5 orders of magnitude (i.e., the E-value of best hit in eukaryotes will be larger than 1 if the best prokaryotic hit E-value is 1E-05). Some HGT genes may have diverged significantly after the HGT event. For example, gene PPERSA_00031570 with an E-value 1E-06 to its best BLAST hit (prokaryotic protein), has a bacterial-like globin (Pfam domain: PF01152) and is therefore highly likely to be of prokaryotes origin. In such cases, an E-value 1E-05 was used if no homolog was found in the eukaryotes. In addition, to confirm the existence of HGT genes, PCR analysis was performed for 20 of 54 HGT genes identified by the bioinformatics (for primers, see Table S4), 100% of which were verified.

### Data access

This Whole Genome Shotgun project has been deposited at DDBJ/EMBL/GenBank under the accession LDAU00000000. The version described in this paper is version LDAU01000000. The raw genome sequences reads have been deposited in Sequence Read Archive (SRA) under accession SRX883501 and SRX883503. Transcriptome data has also been deposited in SRA under accession SRX849480.

## Additional Information

**How to cite this article**: Xiong, J. *et al.* Genome of the facultative scuticociliatosis pathogen *Pseudocohnilembus persalinus* provides insight into its virulence through horizontal gene transfer. *Sci. Rep.* **5**, 15470; doi: 10.1038/srep15470 (2015).

## Supplementary Material

Supplementary Information

## Figures and Tables

**Table 1:** Statistics of *Pseudocohnilembus persalinus* genome and comparison to three other sequenced oligohymenophorean ciliates.

**Species**	***P. persalinus***	***I. multifiliis***	***T. thermophila***	***P. tetraurelia***
Genome size (Mb)	55.5	47.8	103.0	72.1
N50 (Kb)	368	66	521	413
Scaffold number	288	1375	1148	697
Longest scaffold (Mb)	2.0	0.4	2.2	1.0
Sequencing method/platform	Illumina	Sanger/454	Sanger	Sanger
Average GC content	19%	16%	22%	28%
Gene number	13186	8062	26460	39642
Gene density (genes/Mb)	238	169	256	548

*P. persalinus: Pseudocohnilembus persalinus; I. multifiliis: Ichthyophthirius multifiliis; T. thermophila: Tetrahymena thermophila; P. tetraurelia: Paramecium tetraurelia.*

**Table 2:** 54 HGT genes in *Pseudocohnilembus persalinus* genome.

**Gene ID**	**Best hit in NCBI**	**E-vaule**	**Best hit species**	**Species category**	**Function category by annotation**
PPERSA_00056740	Ig family protein	6.00E-31	*Pectobacterium wasabia*	Proteobacteria	Cell adhesion
PPERSA_00029910	Ig family protein	8.00E-23	*Dickeya zeae*	Proteobacteria	Cell adhesion
PPERSA_00047700	Lysophospholipase L1	8.00E-34	*Amycolatopsis azurea*	Actinobacteria	Hemolysis related protein
PPERSA_00098980	phosphatidylinositol-specific phospholipase C1 like protein	3.00E-06	*Flavobacterium sp.*	Bacteroidetes	Hemolysis related protein
PPERSA_00002080	phosphatidylinositol-specific phospholipase C1like protein	8.00E-10	*Streptomyces thermolilacinus*	Actinobacteria	Hemolysis related protein
PPERSA_00035610	hemolysin III family channel protein	1.00E-14	*Gordonia alkanivorans*	Actinobacteria	Hemolysis related protein
PPERSA_00117390	hemopexin repeat-containing protein	4.00E-39	*Flavobacterium beibuense*	Bacteroidetes	Heme related protein
PPERSA_00079580	hemopexin repeat-containing protein	5.00E-45	*Flavobacterium beibuense*	Bacteroidetes	Heme related protein
PPERSA_00031570	hemin receptor	1.00E-06	*Mycobacterium chubuense*	Actinobacteria	Heme related protein
PPERSA_00130810	2OG-Fe(II) oxygenase	3.00E-143	*Aeromonas hydrophila*	Proteobacteria	oxidoreductase
PPERSA_00076120	2OG-Fe(II) oxygenase	8.00E-47	*Kordia algicida*	Bacteroidetes	oxidoreductase
PPERSA_00055830	D-amino acid dehydrogenase small subunit DadA	4.00E-23	*Janthinobacterium sp.*	Proteobacteria	oxidoreductase
PPERSA_00113410	FAD-dependent pyridine nucleotide-disulfide oxidoreductase	0.00E + 00	*Mahella australiensis*	Firmicutes	oxidoreductase
PPERSA_00109590	amine oxidase	6.00E-37	*Microcystis aeruginosa*	Cyanobacteria	oxidoreductase
PPERSA_00073400	NAD-dependent epimerase/dehydratase	3.00E-111	*Nitrosomonas sp.*	Proteobacteria	oxidoreductase
PPERSA_00069080	NAD-dependent epimerase/dehydratase	1.00E-69	*Geobacter uraniireducens*	Proteobacteria	oxidoreductase
PPERSA_00041150	arsenate reductase	2.00E-18	*Pasteurella multocida*	Proteobacteria	oxidoreductase
PPERSA_00069770	glutathione S-transferase	7.00E-21	*Leeia oryzae*	Proteobacteria	/
PPERSA_00059730	major facilitator superfamily MFS_1	2.00E-34	*Clostridium carboxidivorans*	Firmicutes	/
PPERSA_00059750	major facilitator superfamily MFS_1	1.00E-14	*Clostridium drakei*	Firmicutes	/
PPERSA_00084980	magnesium transporter	2.00E-10	*Fischerella muscicola*	Cyanobacteria	/
PPERSA_00107980	MFS-type transporter	6.00E-45	*bacillus massiliosenegalensis*	Firmicutes	/
PPERSA_00125230	Beta-lactamase	6.00E-37	*Paenibacillus sp.*	Firmicutes	/
PPERSA_00036040	glyoxalase/bleomycin resistance protein/dioxygenase	3.00E-36	*Sphingopyxis sp.*	Proteobacteria	/
PPERSA_00036460	thioesterase	2.00E-14	*Desulfomonile tiedjei*	Proteobacteria	/
PPERSA_00036470	thioesterase	3.00E-15	*Alcanivorax hongdengensis*	Proteobacteria	/
PPERSA_00021250	beta-lactamase	5.00E-50	*Cyanothece sp.*	Cyanobacteria	/
PPERSA_00131510	3-oxoacyl-ACP synthase	4.00E-13	*Chlorogloeopsis*	Proteobacteria	/
PPERSA_00117970	DNA polymerase III subunit epsilon	4.00E-18	*Gammaproteobacteria bacterium SCGC AAA003-E02*	Proteobacteria	/
PPERSA_00073390	2-amino-3-ketobutyrate CoA ligase	0.00E + 00	*Candidatus Cloacamonas*	Cloacimonetes	/
PPERSA_00011350	formyl transferase domain protein	3.00E-16	*Streptomyces natalensis*	Actinobacteria	/
PPERSA_00089970	inosine/uridine-preferring nucleoside hydrolase	7.00E-37	*Legionella wadsworthii*	Proteobacteria	/
PPERSA_00125930	bifunctional GMP synthase/glutamine amidotransferase protein	0.00E + 00	*Lentisphaera araneosa*	Chlamydiae	/
PPERSA_00035440	Rhodanese domain protein	5.00E-08	*Flavobacterium sp.*	Bacteroidetes	/
PPERSA_00043810	rhodanese-related sulfurtransferase	1.00E-117	*Endozoicomonas elysicola*	Proteobacteria	/
PPERSA_00057430	SH3 protein, type 3	7.00E-58	*Gemmata obscuriglobus*	Planctomycetes	/
PPERSA_00050710	hypothetical protein	6.00E-12	*Microscilla marina*	Bacteroidetes	/
PPERSA_00036150	putative phosphatase	1.00E-12	*Photobacterium halotolerans*	Proteobacteria	/
PPERSA_00010290	PF08002 family protein	3.00E-13	*Bacteroidetes bacterium*	Bacteroidetes	/
PPERSA_00050920	photopyrone synthase	5.00E-14	*Photorhabdus luminescens*	Proteobacteria	/
PPERSA_00042620	primase	5.00E-07	*Methanosarcina acetivorans*	Methanomicrobia	/
PPERSA_00086310	3-oxoacyl-ACP synthase	7.00E-11	*Cupriavidus sp.*	Proteobacteria	/
PPERSA_00086300	3-oxoacyl-ACP synthase	2.00E-10	*Pseudanabaena sp.*	Cyanobacteria	/
PPERSA_00045220	3-oxoacyl-ACP synthase	2.00E-14	*Granulicella mallensis*	Fibrobacteres	/
PPERSA_00054440	acid phosphatase	1.00E-06	*Flavobacterium sp.*	Bacteroidetes	/
PPERSA_00098990	acid phosphatase	2.00E-06	*Flavobacterium sp.*	Bacteroidetes	/
PPERSA_00121720	rRNA adenine methyltransferase	1.00E-42	*Planktothrix*	Cyanobacteria	/
PPERSA_00076020	radical SAM domain protein	8.00E-81	*Zavarzinella formosa*	Planctomycetes	/
PPERSA_00125500	cytidine deaminase	8.00E-14	*Methanoculleus sp.*	Methanomicrobia	/
PPERSA_00032590	2-hydroxy-6-oxo-6-phenylhexa-2,4-dienoate hydrolase	6.00E-09	*Bordetella avium*	Proteobacteria	/
PPERSA_00117680	membrane-associated protein in eicosanoid and glutathione metabolism (mapeg)	1.00E-16	*Luteimonas mephitis*	Proteobacteria	/
PPERSA_00009640	TPR repeat	4.00E-12	*Microscilla marina*	Bacteroidetes	/
PPERSA_00037920	DTW domain protein	1.00E-30	*Photobacterium damselae*	Proteobacteria	/
PPERSA_00083530	2-nitropropane dioxygenase	5.00E-63	*Kyrpidia tusciae*	Firmicutes	/
