# Supplementary material for: Genome of the facultative scuticociliatosis pathogen Pseudocohnilembus persalinus provides insight into its virulence through horizontal gene transfer
Source: Sci Rep. 2015 Oct 21;5:15470. doi: 10.1038/srep15470 (PMC4614350; doi:10.1038/srep15470)
Supplement: Supplementary Information [file srep15470-s1.pdf]

Genome of the facultative scuticociliatosis pathogen *Pseudocohnilembus persalinus* provides insight into its virulence through horizontal gene transfer

Jie Xiong[1], Guangying Wang[1,2], Jun Cheng[1,3], Miao Tian[1,2], Xuming Pan[4], Alan Warren[5], Chuanqi Jiang[1,2], Dongxia Yuan[1], Wei Miao[1§]

[1]Key Laboratory of Aquatic Biodiversity and Conservation, Institute of Hydrobiology, Chinese Academy of Sciences, Wuhan 430072, China;

[2]University of Chinese Academy of Sciences, Beijing 100049, China

[3]Guangdong Ocean University, Zhanjiang 524088, China

[4]Lab of Protozoology, Department of Biology, College of Life Science and Technology, Harbin Normal University, Harbin 150025, China

[5]Department of Life Sciences, Natural History Museum, Cromwell Road, London SW7 5BD, United Kingdom

[§]Corresponding author: Wei Miao
Email: miaowei@ihb.ac.cn
Tel: +86-27-68780050
Fax: +86-27-68780050

Running title: Horizontal virulence gene transfers in *Pseudocohnilembus persalinus*

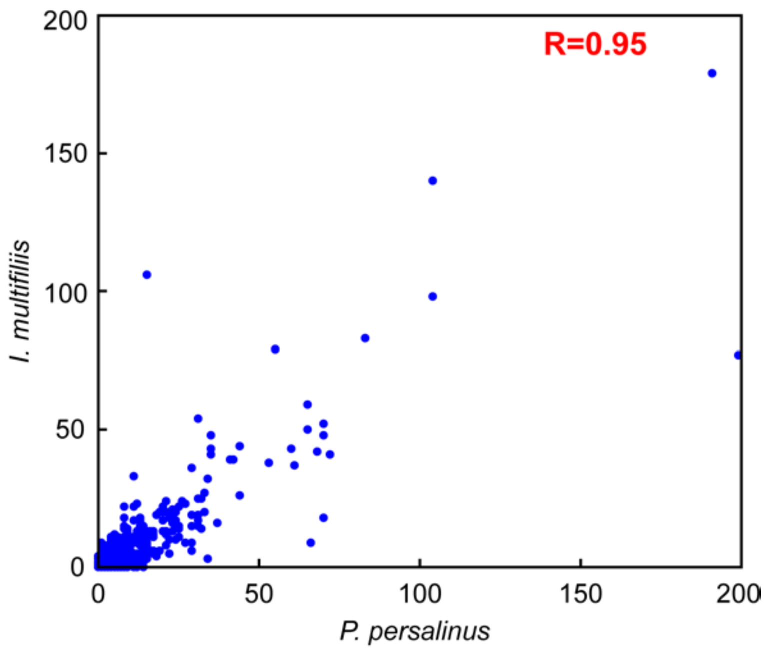

Figure S1. Correlation of the protein domain composition between *I. multifiliis* and *P. persalinus.*Domains were predicted using Pfam database.

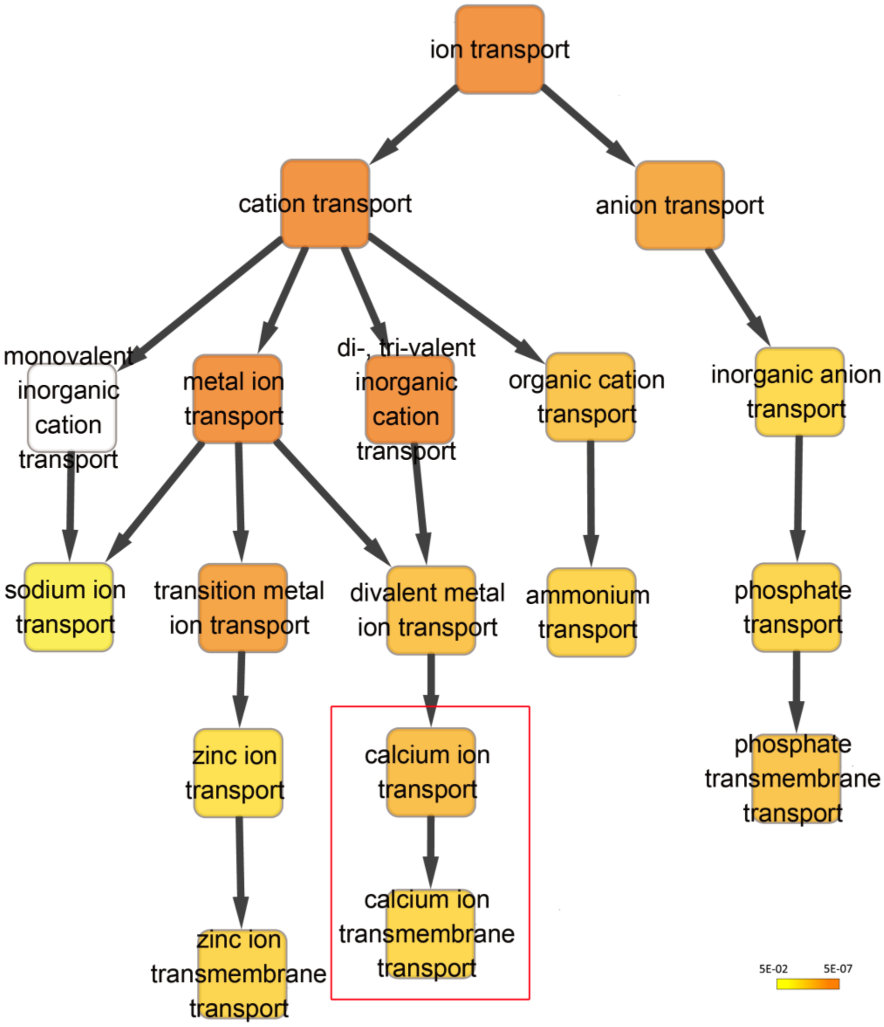

Figure S2. The functional enrichment of specific transporters in *P. persalinus*.Yellow boxes depict the significantly enriched GO terms, calcium-related processes are highlighted with a red box. GO: gene ontology.

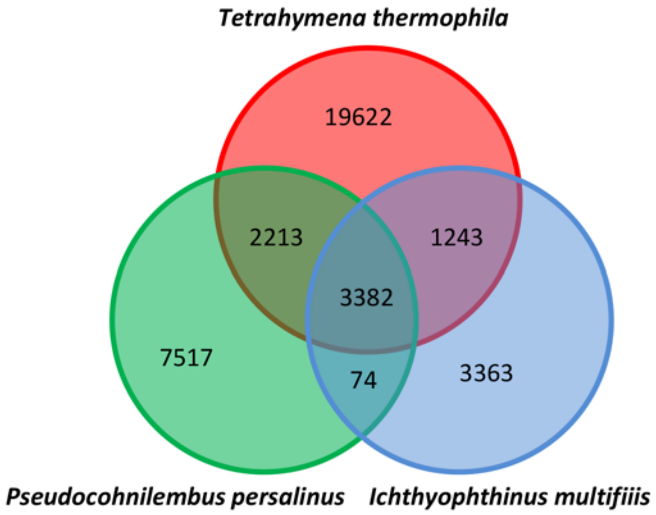

Figure S3. Orthologs among *T. thermophila*, *I. multifiliis* and *P. persalinus*.

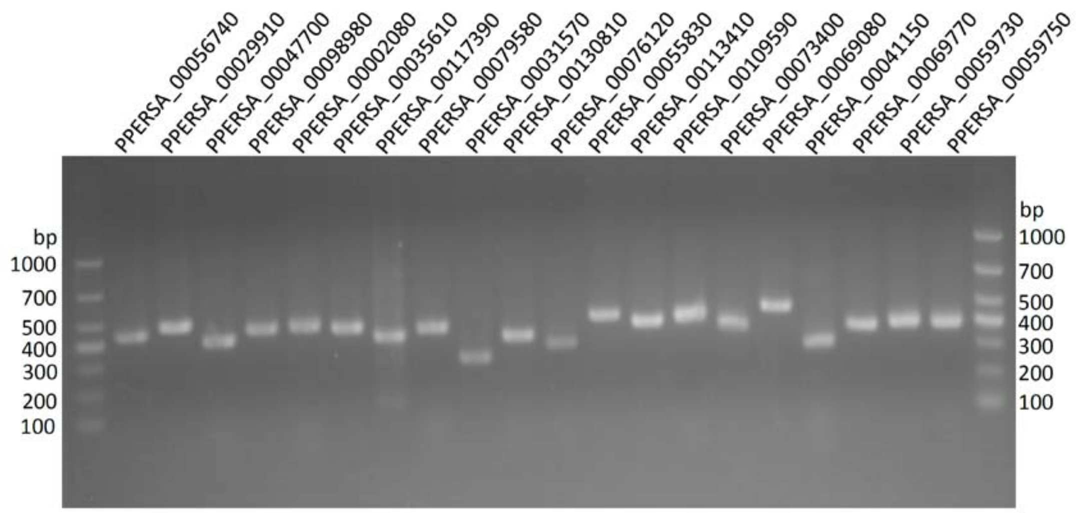

Figure S4. PCR verification of HGT genes.

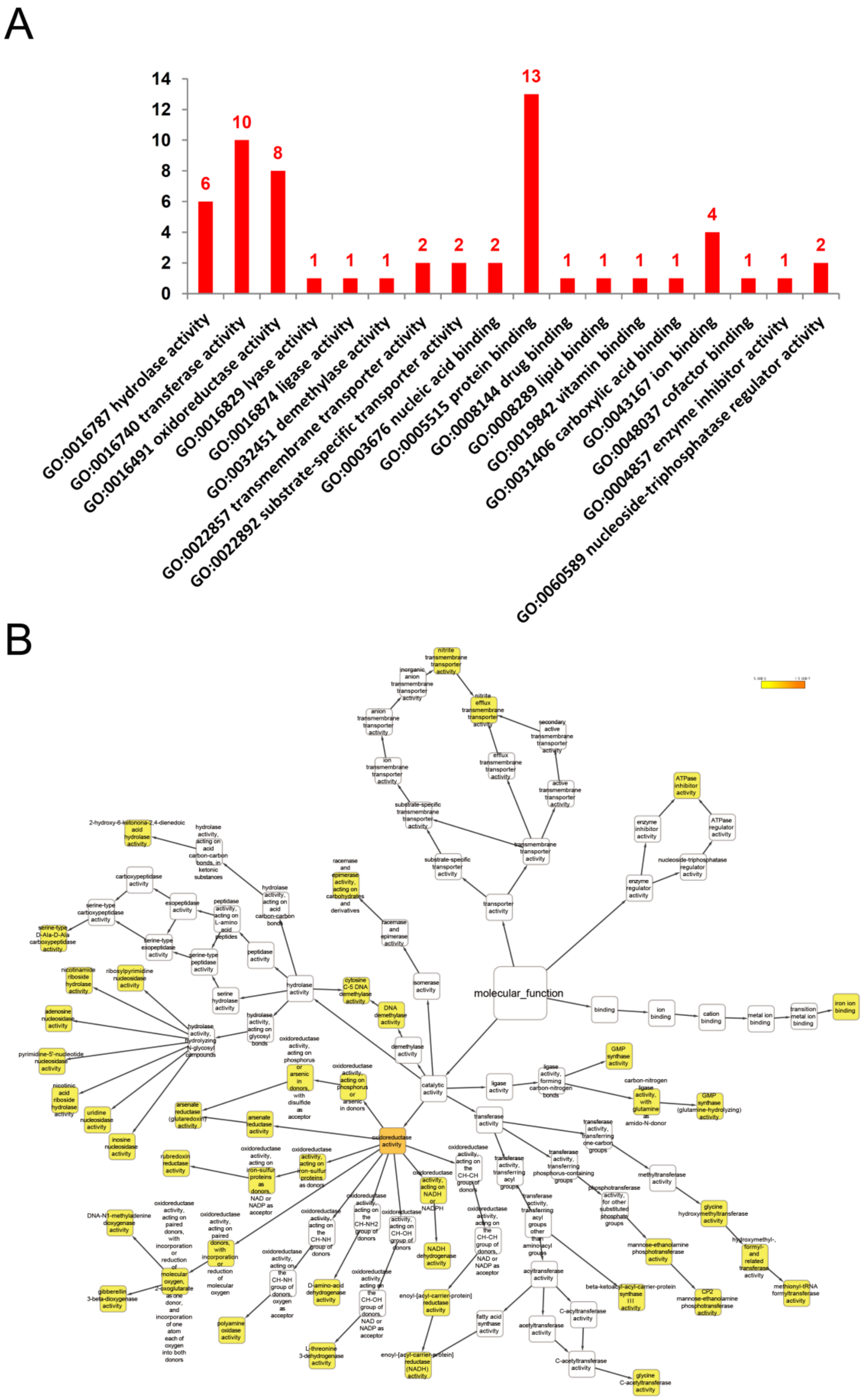

Figure S5. The functional category and enrichment of 54 HGT genes. A, functional categories according to the level 3 GO information; B, an enrichment of 54 HGT genes; yellow boxes are the significantly enriched GO terms. The molecular function of GO was used in both panels. GO: gene ontology.

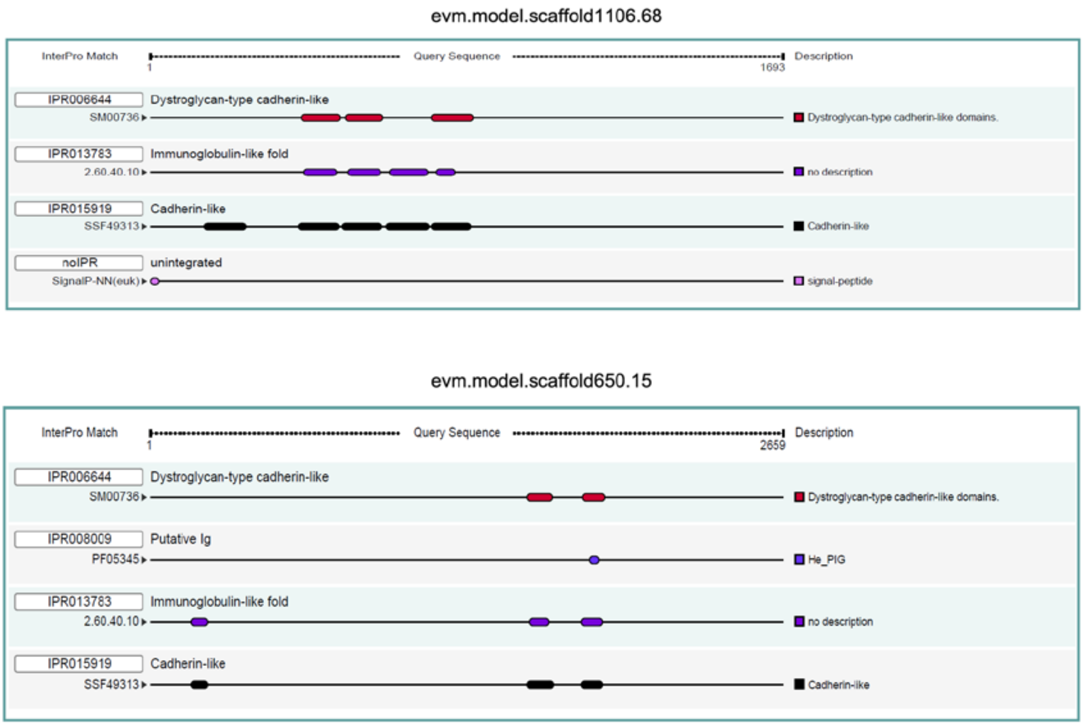

Figure S6. The domain composition of two Ig family HGT genes. Domains were predicted using Interproscan. Interproscan is an integrated predicted tools for many domain knowledge sources; different tracks represent the results predicted by different sources.

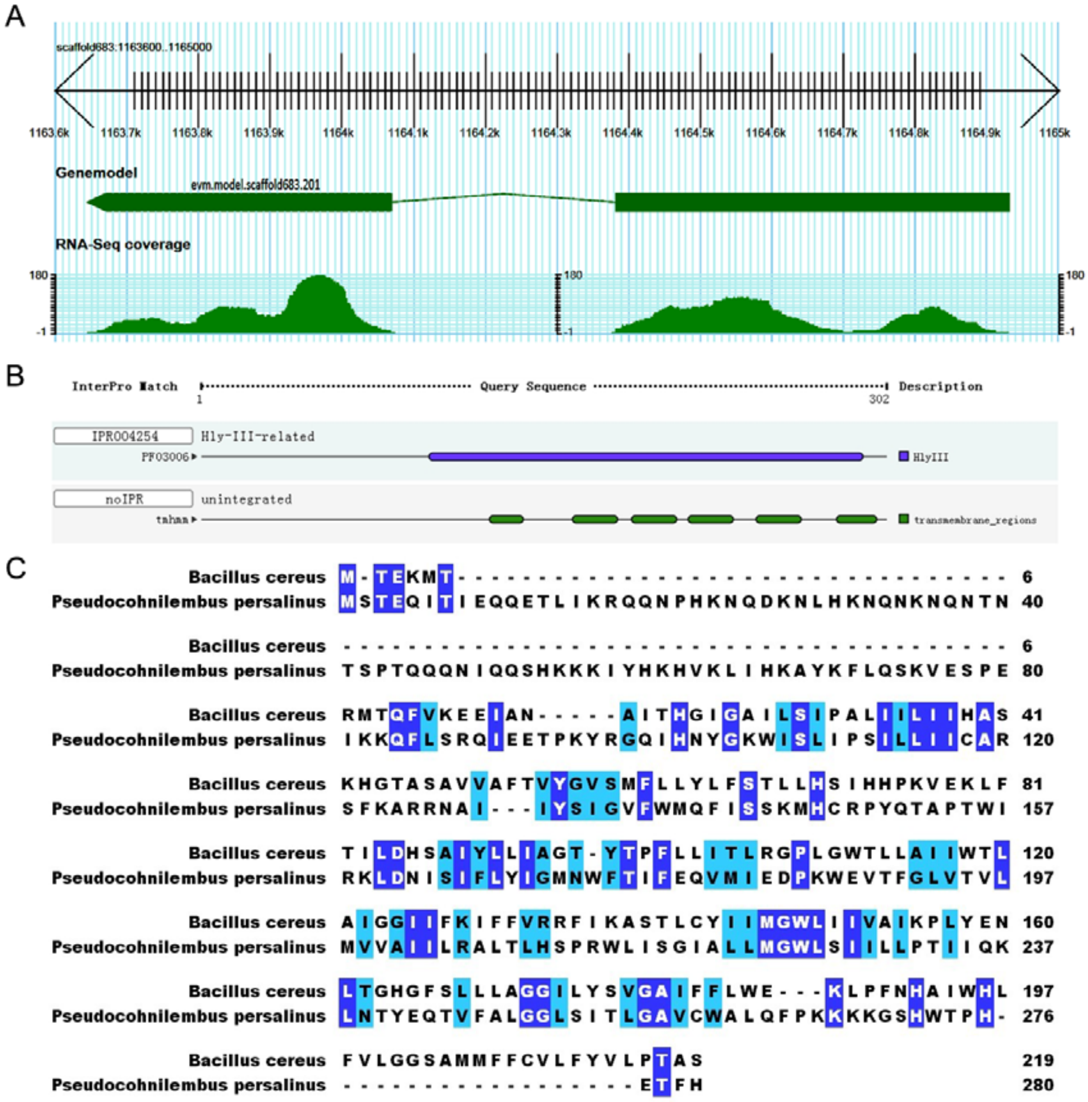

Figure S7. The structure, domain and sequence of the hemolysin III gene. A, the gene structure, one RNA-Seq-supported intron was found in the hemolysin III HGT gene; B, domain and transmembrane helices information; C, sequence comparison between the *P. persalinus*and *Bacillus cereus* (bacterial) hemolysin III gene.

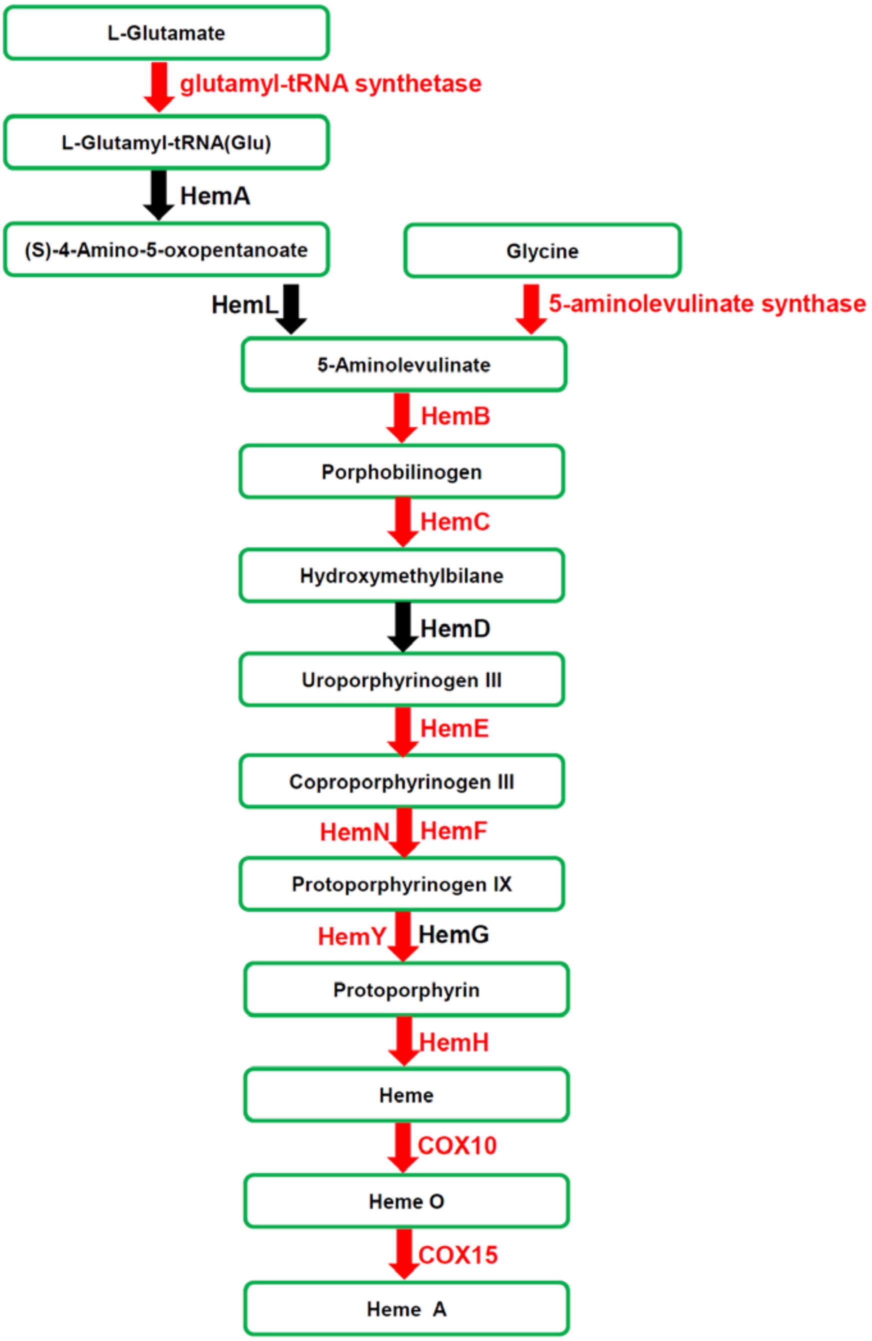

Figure S8. The heme synthesis pathway in *P. persalinus*.The pathway is annotated by mapping the predicted protein sequences to KEGG orthologs. Green boxes represent the compounds. Red arrows indicate the existence of KEGG orthologs (enzymes) in*P. persalinus*. KEGG: Kyoto Encyclopedia of Genes and Genomes.

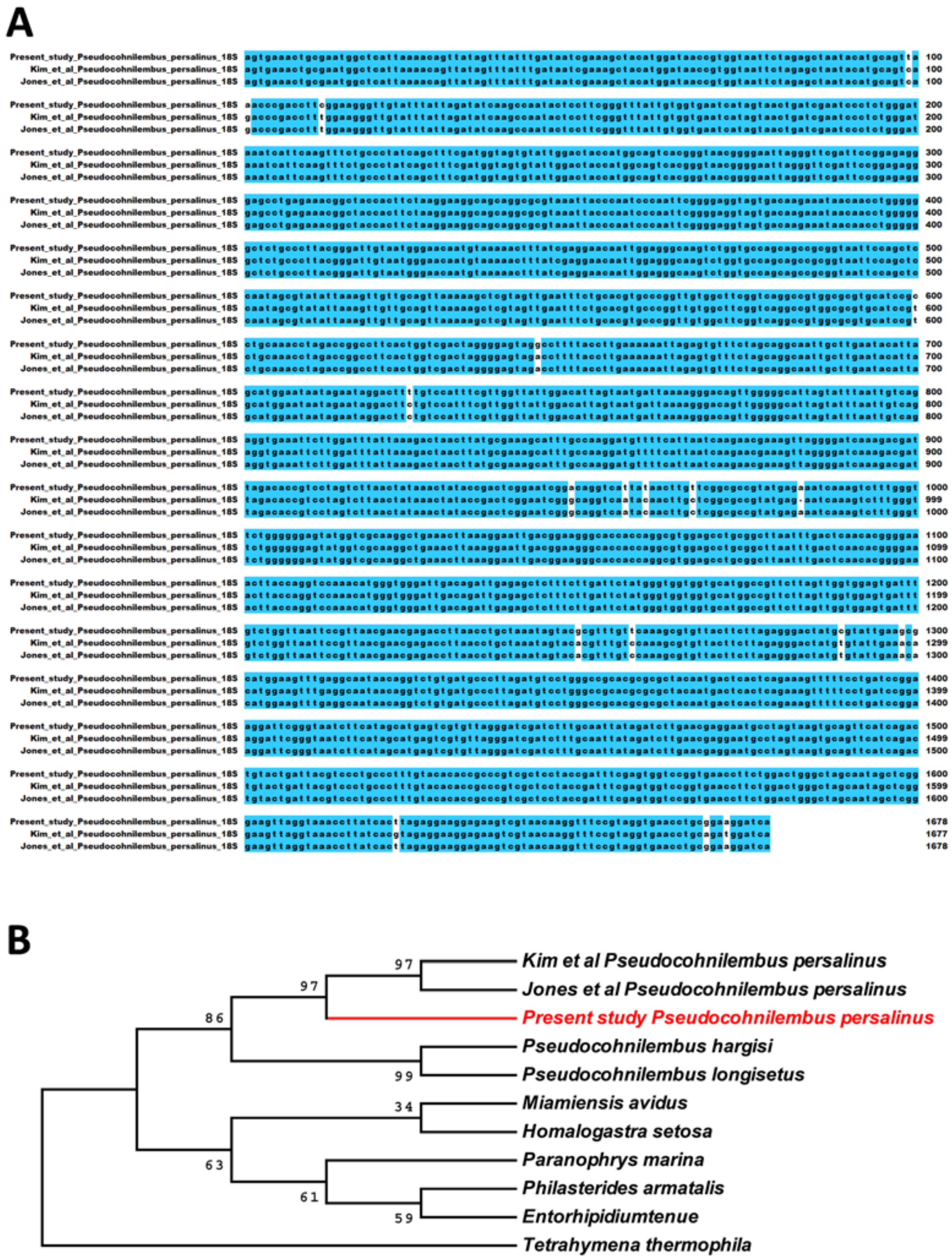

Figure S9. Species Identification of *P. persalinus*.A, sequence alignment of the 18S rDNA in the present study and two previous studies; B, the 18s rDNA gene tree (ML method, 100 bootstrap), showing the close relationship between the present and two previously sequenced isolates of *P. persalinus*.

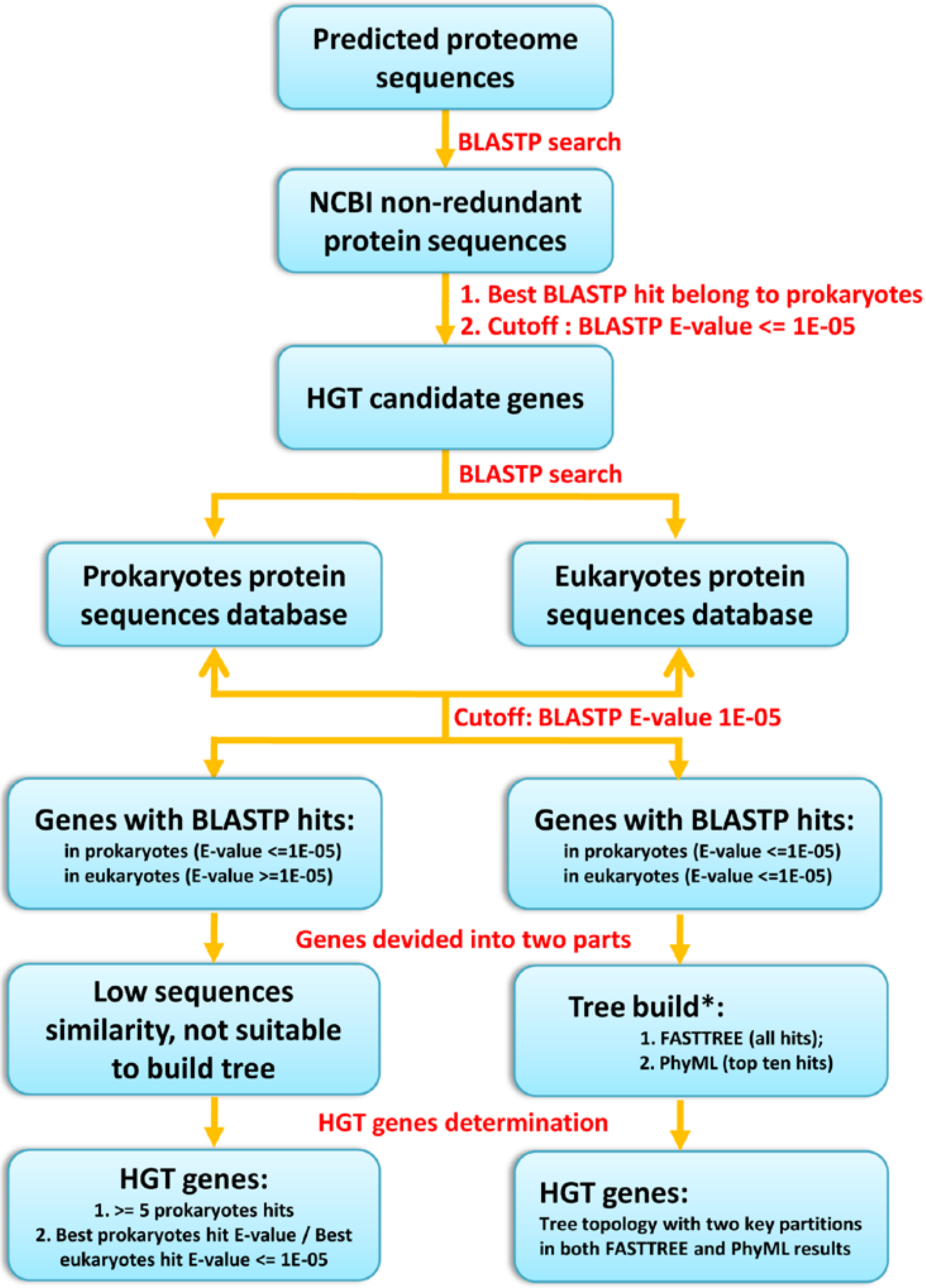

Figure S10. HGT genes identification pipeline.

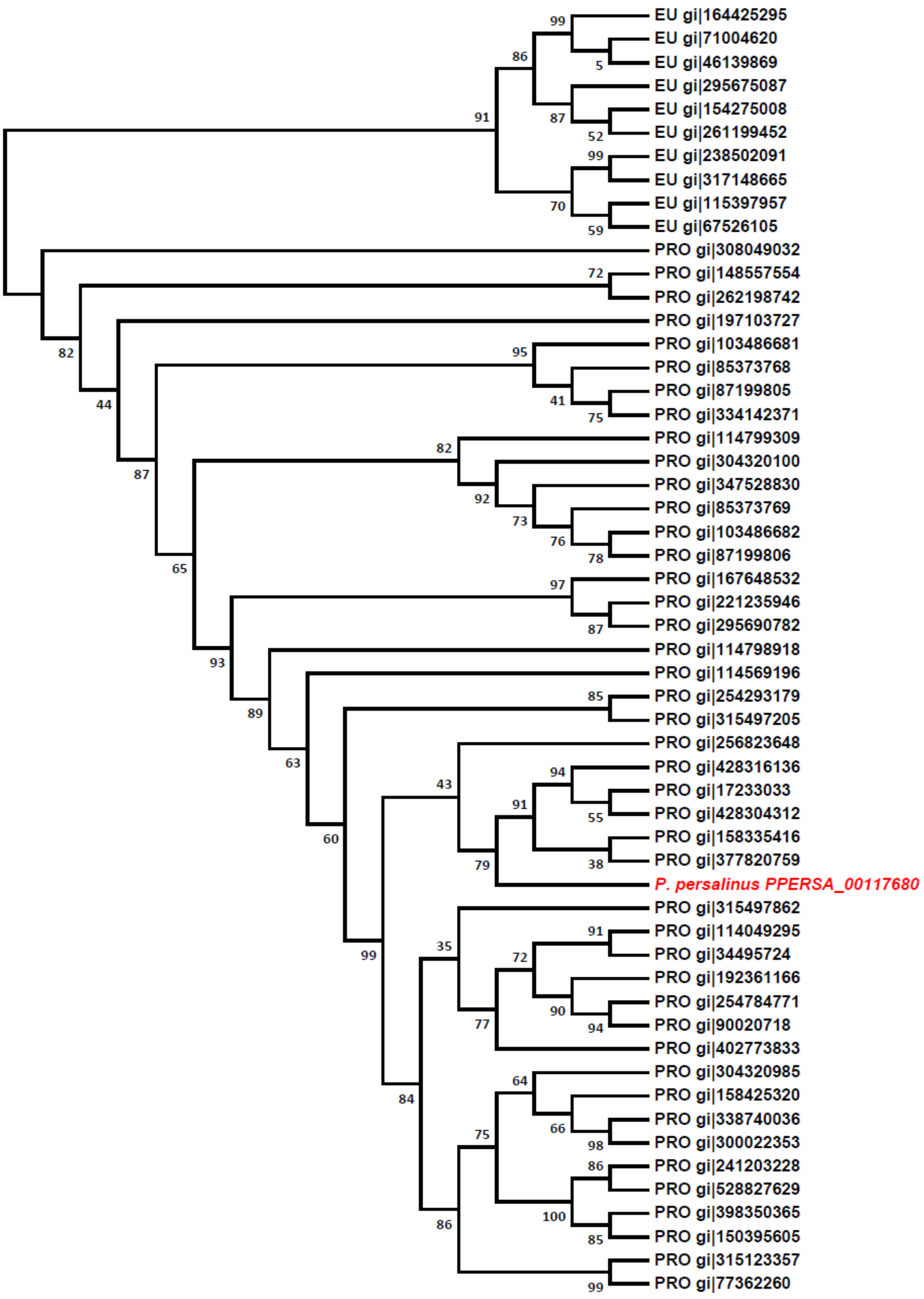

Figure S11. A case (PPERSA_00117680) showed the phylogenetic tree topology used for HGT gene identification. EU, eukaryotes; PRO, prokaryotes. Genes used to construct the phylogenetic tree are indicated by the Genbank gi number. *P. persalinus* gene was highlighted as red.

Table S1. Comparision of the proteases of four sequenced ciliates.

Species	Catalytic class						Percentage of the proteome
	Aspartic	Cysteine	Metallo	Serine	Threonine	Total	
P. persalinus	13	137	113	92	14	369	2.8
I. multifiliis	14	81	119	25	15	254	3.1
P. tetraurelia	48	225	168	95	42	578	1.5
T. thermophila	43	211	139	73	14	480	1.7

Table S2. The 106 *P. persalinus*specific transporters comparing to *T. thermophila*.

Gene_ID	TCDB_best_hit	E-value	TMHMM	Toppred	SCAMPI	TCDB family
PPERSA_00016260	P08104, sodium channel protein, brain iii alpha subunit	3.00E-53	16	22	20	1.A.1.10.1
PPERSA_00025690	O60840, Voltage-dependent L-type calcium channel subunit alpha-1F	2.00E-05	7	6	7	1.A.1.11.11
PPERSA_00035650	Q61290,Voltage-dependent R-type calcium channel alpha-1e subunit	2.00E-05	3	3	4	1.A.1.11.3
PPERSA_00099750	Q61290,Voltage-dependent R-type calcium channel alpha-1e subunit	2.00E-19	3	4	4	1.A.1.11.3
PPERSA_00125210	Q61290,Voltage-dependent R-type calcium channel alpha-1e subunit	5.00E-22	6	7	6	1.A.1.11.3
PPERSA_00034770	Q61290,Voltage-dependent R-type calcium channel alpha-1e subunit	5.00E-30	9	9	10	1.A.1.11.3
PPERSA_00026650	Q61290,Voltage-dependent R-type calcium channel alpha-1e subunit	8.00E-06	9	10	10	1.A.1.11.3
PPERSA_00107800	Q8R4F0, Mucolipin-3	3.00E-07	5	6	6	1.A.5.3.2
PPERSA_00042560	Q8IZK6, Mucolipin-2	3.00E-04	2	4	3	1.A.5.3.3
PPERSA_00111700	Q8IZK6, Mucolipin-2	9.00E-07	5	8	7	1.A.5.3.3
PPERSA_00003520	Q02563, Synaptic vesicle protein 2 (SV2)	3.00E-09	6	6	6	2.A.1.22.1
PPERSA_00028190	Q02563, Synaptic vesicle protein 2 (SV2)	5.00E-19	9	10	9	2.A.1.22.1
PPERSA_00021720	Q02563, Synaptic vesicle protein 2 (SV2)	6.00E-13	12	11	12	2.A.1.22.1
PPERSA_00015020	Q6KCM7, Calcium-binding mitochondrial carrier protein SCaMC-2	1.00E-58	2	3	3	2.A.29.23.1
PPERSA_00013920	Q6KCM7, Calcium-binding mitochondrial carrier protein SCaMC-2	5.00E-75	2	4	3	2.A.29.23.1
PPERSA_00005690	P39109, Metal resistance protein YCF1 (Yeast cadmium factor 1)	2.00E-56	4	4	4	3.A.1.208.11
PPERSA_00027070	P39109, Metal resistance protein YCF1 (Yeast cadmium factor 1)	1.00E-102	3	3	5	3.A.1.208.11
PPERSA_00095340	P16389, Potassium voltage-gated channel subfamily A member 2	6.00E-17	2	3	2	1.A.1.2.10
PPERSA_00041650	P16389, Potassium voltage-gated channel subfamily A member 2	3.00E-06	1	3	3	1.A.1.2.10
PPERSA_00101590	Q02094, Rhesus blood group-associated glycoprotein	3.00E-47	8	9	9	1.A.11.4.3
PPERSA_00101600	Q02094, Rhesus blood group-associated glycoprotein	1.00E-57	12	11	12	1.A.11.4.3
PPERSA_00119880	Q8VZR6, Probable inositol transporter 1	2.00E-82	12	11	10	2.A.1.1.66
PPERSA_00033520	Q86VW1, Solute carrier family 22 member 16	3.00E-14	6	5	6	2.A.1.19.12

PPERSA_00091860	O35633, Vesicular inhibitory amino acid transporter	2.00E-04	6	6	6	2.A.18.5.3
PPERSA_00057540	A7ZUZ0,Melibiose carrier protein	3.00E-05	12	11	10	2.A.2.1.1
PPERSA_00105270	O43808,Peroxisomal membrane protein PMP34 (34 kDaperoxisomal membrane protein) (Solute carrier family 25, member 17)	4.00E-30	2	5	4	2.A.29.20.1
PPERSA_00052210	O04619, Adenine nucleotide transporter, ADNT1	2.00E-15	2	5	2	2.A.29.23.4
PPERSA_00034270	Q00319,Peroxisomal membrane protein PMP47B	8.00E-22	10	11	10	2.A.29.6.1
PPERSA_00037870	P39580 2.A.50.2.1 Protein dltB - Bacillus subtilis.Length = 395	3.00E-06	7	9	8	2.A.50.2.1
PPERSA_00037910	Q9SKZ5, Expressed protein	3.00E-16	4	6	4	2.A.71.2.2
PPERSA_00084580	P11024, NAD(P) Transhydrogenase, mitochondrial precursor (EC: 1.6.1.2) (pyridine nucleotide transhydrogenase) (nicotinamide nucleotide transhydrogenase)	0	11	12	10	3.D.2.3.1
PPERSA_00089600	P21439 3.A.1.201.3 Multidrug resistance protein 3 (P-glycoprotein 3) - Homo sapiens (Human).Length = 1286	2.00E-97	6	5	5	3.A.1.201.3
PPERSA_00105470	O22526,Cation-chloride co-transporter	5.00E-96	9	12	13	2.A.30.6.1
PPERSA_00046210	P54144, High affinity ammonium transporter	7.00E-85	11	12	11	1.A.11.2.1
PPERSA_00120960	Q8RXN0, White-brown complex homolog protein 11	5.00E-70	7	5	6	3.A.1.204.8
PPERSA_00057820	P54144, High affinity ammonium transporter	4.00E-65	8	10	8	1.A.11.2.1
PPERSA_00057690	O07488, Sulfate permease	3.00E-64	9	9	11	2.A.53.3.1
PPERSA_00049890	P35498, Sodium channel protein type 1 subunit alpha - Homo sapiens (Human).Length = 2009	2.00E-62	16	19	15	1.A.1.10.7
PPERSA_00063790	O59666, Copper-transporting ATPase ccc2	1.00E-57	8	8	8	3.A.3.5.29
PPERSA_00021470	Q71RS6, Sodium/potassium/calcium exchanger 5	1.00E-56	8	10	9	2.A.19.4.6
PPERSA_00039930	P08716,Hemolysin secretion ATP-binding protein	2.00E-53	1	3	1	3.A.1.109.1
PPERSA_00113390	Q9SVT8, Ammonium transporter 1 member 4	5.00E-48	8	10	9	1.A.11.2.6
PPERSA_00120950	Q8RXN0, White-brown complex homolog protein 11	4.00E-46	7	6	6	3.A.1.204.8
PPERSA_00027040	Q7GB25, ABC transporter C family member 5	3.00E-40	1	1	1	3.A.1.208.22
PPERSA_00034780	O95180, Voltage-dependent T-type calcium channel subunit alpha-1H	4.00E-40	6	7	7	1.A.1.11.5
PPERSA_00092040	Q86UD5, NHEDC2 protein	8.00E-40	11	10	10	2.A.36.8.1
PPERSA_00063060	Q38954, Putative phosphate permease	9.00E-38	10	12	11	2.A.20.2.4

PPERSA_00114070	O43772, Mitochondrial carnitine/acylcarnitine carrier protein	4.00E-37	2	4	3	2.A.29.8.3
PPERSA_00079800	Q9CR58,Similar to brain mitochondrial carrier protein-1 (BMCP-1)	2.00E-36	2	3	2	2.A.29.24.2
PPERSA_00094200	Q8NEW0, Zinc transporter ZnT-7	5.00E-36	10	10	10	2.A.4.4.5
PPERSA_00044240	Q8RXN0, White-brown complex homolog protein 11	5.00E-36	4	4	4	3.A.1.204.8
PPERSA_00059080	Q38954, Putative phosphate permease	4.00E-34	11	12	11	2.A.20.2.4
PPERSA_00021960	Q8S403, Putative phosphate transporter 1	2.00E-33	4	4	4	2.A.94.1.1
PPERSA_00042340	A0ZXK6, Monosaccharide transporter	3.00E-28	10	12	10	2.A.1.1.43
PPERSA_00039140	Q5I0E9, Multidrug and toxin extrusion protein 1	4.00E-28	11	11	10	2.A.66.1.15
PPERSA_00110210	P35670, Copper-transporting atpase 2 (ec 3.6.3.4) (copper pump 2)	4.00E-26	3	3	3	3.A.3.5.3
PPERSA_00130700	Q5BL44, Sodium-dependent phosphate transporter 1	2.00E-25	12	12	12	2.A.20.2.6
PPERSA_00097460	Q6L8F3, Zinc transporter LIV1	1.00E-23	8	8	6	2.A.5.4.2
PPERSA_00101950	P23500 , Mitochondrial RNA splicing protein MRS4	1.00E-22	4	6	3	2.A.29.5.2
PPERSA_00123340	Q9ULF5, Zinc transporter ZIP10	1.00E-21	7	8	7	2.A.5.4.6
PPERSA_00119980	Q15070, Mitochondrial inner membrane protein OXA1L	1.00E-21	3	4	3	2.A.9.1.2
PPERSA_00104800	P32386, ATP-dependent bile acid permease	8.00E-21	1	2	1	3.A.1.208.12
PPERSA_00060790	Q6PML9, Zinc transporter 9	3.00E-19	4	4	4	2.A.4.6.1
PPERSA_00119740	Q93IP6, Ammonium/methylammoniumpermease	6.00E-19	11	10	9	1.A.11.2.3
PPERSA_00124860	A0ZXK6, Monosaccharide transporter	3.00E-17	7	10	8	2.A.1.1.43
PPERSA_00097710	Q86UD5, NHEDC2 protein	3.00E-17	4	4	5	2.A.36.8.1
PPERSA_00102210	Q7Z3S7,Voltage-dependent calcium channel subunit alpha-2/delta-4	1.00E-16	1	1	1	8.A.18.4.1
PPERSA_00076980	A1Z264, Sugar/H+ symporter	1.00E-15	10	9	8	2.A.1.1.69
PPERSA_00011330	P02982, Tetracycline resistance protein, class A	1.00E-15	10	8	9	2.A.1.2.4
PPERSA_00051310	Q9JM15, Neuronal glutamine transporter	3.00E-15	11	10	9	2.A.18.6.1
PPERSA_00093400	O15554, Intermediate conductance calcium-activated potassium channel protein 4	3.00E-13	6	6	6	1.A.1.16.2
PPERSA_00028830	P21817, Ryanodine receptor 1	5.00E-13	5	7	6	1.A.3.1.2
PPERSA_00092060	A0L3E7, Chromate transporter, chromate ion transporter (CHR) family	5.00E-12	8	11	8	2.A.51.1.4

PPERSA_00094760	Q9VNJ5, Protein dispatched	3.00E-11	12	12	14	2.A.6.9.1
PPERSA_00041810	P38767, Hypothetical 64.2 kda protein in slt2-put2 intergenic region	4.00E-11	2	3	3	2.A.66.1.5
PPERSA_00126160	Q86GI9, KVS-1 - Caenorhabditiselegans	8.00E-11	2	2	2	1.A.1.2.8
PPERSA_00117020	O07488, Sulfate permease	2.00E-10	11	9	10	2.A.53.3.1
PPERSA_00098510	P23631, Alpha-latrotoxin precursor	3.00E-10	1	1	1	1.C.63.1.1
PPERSA_00081160	Q8NTX0,Permeases of the major facilitator superfamily	3.00E-10	3	4	3	2.A.1.1.53
PPERSA_00007270	Q8NEW0, Zinc transporter ZnT-7	2.00E-09	6	6	7	2.A.4.4.5
PPERSA_00097360	P0AAC4, Inner membrane protein ybhL	2.00E-08	5	6	5	9.B.24.2.2
PPERSA_00023360	Q95V25, Calcium-activated potassium channel slo-1	3.00E-08	3	3	4	1.A.1.3.3
PPERSA_00048800	P38921, Putative mitochondrial carrier protein PET8	4.00E-08	4	4	3	2.A.29.18.1
PPERSA_00091790	Q9LDC0, 42 kDa peptidyl-prolyl isomerase	4.00E-08	1	1	1	8.A.11.1.1
PPERSA_00071890	Q9KRU4, Probable multidrug resistance protein norM (Na(+)/drug antiporter)	8.00E-08	6	6	5	2.A.66.1.2
PPERSA_00006010	Q5BL44, Sodium-dependent phosphate transporter 1	9.00E-08	13	13	13	2.A.20.2.6
PPERSA_00108680	Q9H0U3, Implantation-associated protein	1.00E-07	3	3	3	9.A.45.1.1
PPERSA_00057310	O24662, Aquaporin 1	2.00E-07	2	5	5	1.A.8.11.2
PPERSA_00100560	P73948, Slr1508 protein	3.00E-07	1	2	2	2.A.38.4.5
PPERSA_00045830	Q9UL62, Short transient receptor potential channel 5	2.00E-06	3	5	3	1.A.4.1.7
PPERSA_00094520	Q9FKS8, Lysine histidine transporter 1	2.00E-06	7	6	6	2.A.18.2.2
PPERSA_00114840	Q45308,Grma protein	6.00E-06	2	3	2	2.A.37.2.3
PPERSA_00094320	Q96QE2, Proton myo-inositol co-transporter (Hmit)	8.00E-06	6	5	5	2.A.1.1.25
PPERSA_00099270	P08995, Nodulin-26 (n-26)	1.00E-05	6	7	4	1.A.8.12.1
PPERSA_00038860	Q9VHS6, CG7459-PA	2.00E-05	3	3	3	1.A.56.1.7
PPERSA_00068860	A9QMN9, TPK1	3.00E-05	3	3	3	1.A.1.7.4
PPERSA_00107980	Q92339, High-affinity gluconate transporter ght3 (Hexose transporter 3)	4.00E-05	11	12	11	2.A.1.1.23
PPERSA_00116400	Q8WPZ6,Aquaglyceroporin	6.00E-05	6	6	6	1.A.8.14.1
PPERSA_00047490	O29993, Iron (II) transporter (FeoB-1)	8.00E-05	1	2	2	9.A.8.1.8

PPERSA_00130660	Q9FGL2, Aquaporin TIP2-3	1.00E-04	1	1	3	1.A.8.10.4
PPERSA_00014350	P94131,Cis,cis-muconate transport protein	1.00E-04	12	12	10	2.A.1.15.4
PPERSA_00040270	Q86G79, Arginine transporter AAP3	1.00E-04	3	3	3	2.A.18.6.9
PPERSA_00080590	P97260,Srebp cleavage activating protein	1.00E-04	5	8	7	2.A.6.6.4
PPERSA_00115630	Q37050, Chloroplast envelope membrane protein	1.00E-04	1	1	1	9.B.73.1.1
PPERSA_00079020	Q07711, DNA topoisomerase I (EC 5.99.1.2)	2.00E-04	1	2	1	3.A.7.7.1
PPERSA_00079050	Q6INM0, MGC82368 protein	3.00E-04	1	3	2	1.C.39.3.2

Note that all the transporters were annotated and assign the five TCDB characters class, specific transporters means that that the class (represent by the five characters) was only found in *P. persalinus* but not *T. thermophila*. For TMHMM, Toppred and SCAMPI, the number of predicted transmembrane helix listed.

Table S3. 74 HGT genes in*T.thermophila*.

Gene_ID	**Annotation**	**E-value**	**Function category**
TTHERM_00628500	(myosin heavy-chain) kinase [Haliscomenobacterhydrossis DSM 1100]	1.00E-127	Protein kinase
TTHERM_00439260	(myosin heavy-chain) kinase [Haliscomenobacterhydrossis DSM 1100]	6.00E-95	Protein kinase
TTHERM_01006570	(myosin heavy-chain) kinase [Haliscomenobacterhydrossis DSM 1100]	1.00E-162	Protein kinase
TTHERM_00741670	(myosin heavy-chain) kinase [Haliscomenobacterhydrossis DSM 1100]	1.00E-135	Protein kinase
TTHERM_00701140	serine/threonine protein kinase [Herpetosiphonaurantiacus DSM 785]	9.00E-13	Protein kinase
TTHERM_01371780	serine/threonine protein kinase [Mycobacterium parascrofulaceum]	6.00E-09	Protein kinase
TTHERM_01131810	chemotaxis sensory transducer [Pseudomonas syringaepv. aptata str. DSM 50252]	2.00E-12	chemotaxis protein
TTHERM_00125780	chemotaxis sensory transducer [Pseudomonas syringaepv. syringae B728a]	1.00E-10	chemotaxis protein
TTHERM_01131800	chemotaxis sensory transducer, partial [Pseudomonas syringaepv. japonica str. M301072]	4.00E-11	chemotaxis protein
TTHERM_01050500	chemotaxis protein [Pseudomonas coronafaciens]	5.00E-11	chemotaxis protein
TTHERM_00126830	chemotaxis protein [Pseudomonas coronafaciens]	3.00E-08	chemotaxis protein
TTHERM_01050490	chemotaxis protein [Pseudomonas coronafaciens]	6.00E-12	chemotaxis protein
TTHERM_01050480	chemotaxis protein [Pseudomonas coronafaciens]	6.00E-12	chemotaxis protein
TTHERM_00126840	chemotaxis protein [Pseudomonas coronafaciens]	4.00E-09	chemotaxis protein
TTHERM_01130780	chemotaxis protein [Pseudomonas syringae group genomosp. 3]	3.00E-10	chemotaxis protein
TTHERM_00324390	methyl-accepting chemotaxis protein [Bacillus halodurans C-125]	6.00E-08	chemotaxis protein
TTHERM_00191490	methyl-accepting chemotaxis protein [Clostridium beijerinckii]	5.00E-07	chemotaxis protein
TTHERM_00125790	methyl-accepting chemotaxis protein [Pyrococcusyayanosii CH1]	1.00E-08	chemotaxis protein
TTHERM_00125800	methyl-accepting chemotaxis protein [Pyrococcusyayanosii CH1]	2.00E-09	chemotaxis protein
TTHERM_00947350	methyl-accepting chemotaxis sensory transducer [Thermotogapetrophila RKU-1]	1.00E-05	chemotaxis protein
TTHERM_00125819	putative methyl-accepting chemotaxis protein [Vibrio azureus]	3.00E-08	chemotaxis protein
TTHERM_00361630	Tetratricopeptide repeat family [Coleofasciculuschthonoplastes]	4.00E-41	TPR family protein

TTHERM_00697150	tetratricopeptide repeat protein [Desulfobacterpostgatei]	2.00E-10	TPR family protein
TTHERM_00778410	tetratricopeptide repeat protein [Methanoregulaformicica SMSP]	1.00E-142	TPR family protein
TTHERM_01001510	tetratricopeptide repeat protein [Prevotella sp. CAG:1031]	9.00E-23	TPR family protein
TTHERM_01014690	tetratricopeptide repeat protein, partial [Porphyromonasgingivalis]	1.00E-12	TPR family protein
TTHERM_00697260	Tetratricopeptide TPR_2 repeat-containing protein [Caldithrixabyssi]	6.00E-11	TPR family protein
TTHERM_00815110	TPR repeat [Methanosarcinabarkeri str. Fusaro]	1.00E-37	TPR family protein
TTHERM_00283270	TPR repeat [Methanosarcinabarkeri str. Fusaro]	5.00E-49	TPR family protein
TTHERM_00701190	TPR repeat containing protein-like protein [Adinetavaga]	3.00E-18	TPR family protein
TTHERM_00657580	TPR repeat containing protein-like protein [Philodinaroseola]	3.00E-53	TPR family protein
TTHERM_00079990	TPR repeat domain-containing protein [Anabaena sp. 90]	3.00E-48	TPR family protein
TTHERM_00617751	TPR repeat domain-containing protein [Anabaena sp. 90]	2.00E-24	TPR family protein
TTHERM_00188800	TPR repeat domain-containing protein [Anabaena sp. 90]	6.00E-18	TPR family protein
TTHERM_00346760	TPR repeat family protein [Lyngbyaaestuarii]	1.00E-12	TPR family protein
TTHERM_00219440	D-alanyl-D-alanine carboxypeptidase family protein [Winogradskyellapsychrotolerans]	1.00E-52	/
TTHERM_00348900	DNA alkylation repair enzyme [Pseudomonas chlororaphis]	9.00E-64	/
TTHERM_00348890	DNA alkylation repair enzyme [Pseudomonas sp. GM50]	5.00E-81	/
TTHERM_00122050	FAD-binding protein [Aeromonasveronii]	1.00E-155	/
TTHERM_00784420	glycine cleavage system H protein [Haliscomenobacterhydrossis DSM 1100]	3.00E-10	/
TTHERM_00245770	glycoside hydrolase family 5 [Gillisialimnaea]	1.00E-117	/
TTHERM_00312470	glycosyltransferase family 2 [Oscillatorianigro-viridis PCC 7112]	4.00E-08	/
TTHERM_01227790	GNAT family acetyltransferase [Solobacteriummoorei]	9.00E-18	/
TTHERM_00348080	hypothetical protein LEUM_1748 [Leuconostocmesenteroides subsp. mesenteroides ATCC 8293]	4.00E-09	/
TTHERM_00325600	membrane protein [Vibrio coralliilyticus]	3.00E-14	/
TTHERM_00486540	membrane protein [Vibrio splendidus]	2.00E-30	/
TTHERM_00218950	metal-dependent hydrolase [Modestobactermarinus]	5.00E-15	/
TTHERM_00348790	metal-dependent hydrolase [Rhizobium sp. CF142]	2.00E-10	/

TTHERM_00592830	NAD-binding domain 4 [Capnocytophagacanimorsus Cc5]	3.00E-27	/
TTHERM_00227539	NB-ARC domain-containing protein [Cyanothece sp. PCC 7822]	5.00E-20	/
TTHERM_00170580	NB-ARC domain-containing protein [Cyanothece sp. PCC 7822]	8.00E-38	/
TTHERM_00486460	nucleotidyltransferase [Enterobacter sp. R4-368]	2.00E-12	/
TTHERM_00194420	peptidase S1 and S6 chymotrypsin/Hap [Cyanothece sp. PCC 8802]	4.00E-28	/
TTHERM_00833880	peptidase S1 and S6 chymotrypsin/Hap [Cyanothece sp. PCC 8802]	2.00E-33	/
TTHERM_00107040	peptidase-like protein [Trichodesmiumerythraeum IMS101]	5.00E-83	/
TTHERM_00219050	peptide methionine sulfoxide reductase [Rhodopirellulamaiorica]	1.00E-72	/
TTHERM_01029920	photorhabdus clumping factor C [Photorhabdustemperata subsp. thracensis]	1.00E-25	/
TTHERM_01263960	predicted protein [Hordeumvulgare subsp. vulgare]	9.00E-39	/
TTHERM_01174650	PREDICTED: protein NLRC3 [Otolemurgarnettii]	2.00E-30	/
TTHERM_00325620	protein of unknown function DUF6 transmembrane [Stanieriacyanosphaera PCC 7437]	1.00E-28	/
TTHERM_00689910	putative nucleotidyltransferase [Pseudoalteromonasluteoviolacea]	1.00E-06	/
TTHERM_00437750	putative O-linked N-acetylglucosaminetransferase, SPINDLY family [Synechococcus sp. PCC 7502]	3.00E-10	/
TTHERM_00538920	putative Sarcosine oxidase [Legionella pneumophila subsp. pneumophila]	4.00E-09	/
TTHERM_00408900	putative uncharacterized protein [Eubacterium sp. CAG:146]	7.00E-64	/
TTHERM_00408930	putative uncharacterized protein [Eubacterium sp. CAG:146]	4.00E-55	/
TTHERM_00520910	ribonuclease R [Tannerella forsythia ATCC 43037]	1.00E-05	/
TTHERM_01232190	structure-specific endonuclease subunit SLX4 [Cricetulusgriseus]	4.00E-13	/
TTHERM_00678120	sucrose-phosphate phosphatase subfamily [Spirochaetasmaragdinae DSM 11293]	3.00E-08	/
TTHERM_00214670	surface antigen BspA [Treponemaazotonutricium ZAS-9]	6.00E-11	/
TTHERM_01192460	type IV pilus biogenesis/stability protein PilW [Lyngbyaaestuarii]	2.00E-15	/
TTHERM_00227570	WD40 repeat-containing protein [Nostoc sp. PCC 7524]	1.00E-19	/
TTHERM_01351030	WD40 repeat-containing protein [Nostoc sp. PCC 7524]	7.00E-53	/
TTHERM_01045800	alpha/beta hydrolase fold protein [Chlorobiumphaeovibrioides DSM 265]	2.00E-07	/
TTHERM_00431240	Appr-1-p processing domain protein [Aciduliprofundumboonei T469]	1.00E-09	/

TTHERM_00227390	cell division control protein 48 AAA family protein [Methanosarcinaacetivorans C2A]	1.00E-11	/

Table S4. Primers used for the HGT genes verification.

HGT_Gene_ID	F_primer_5'-3'	R_primer_5'-3'	Product_length
PPERSA_00056740	TGTCTACGGAATCTTAATTG	TCTTGAAACAACTCAAACACT	423
PPERSA_00029910	CAATTTTAACGAATTTCCAGC	AGGACCAGTTAAGATTAAAA	461
PPERSA_00047700	GGGGTTAACGATATTATTGA	AAAATTATCTTTACGGAAGG	380
PPERSA_00098980	AGGTCAGGGTATTTTAAGTT	TTATAAAATTTGGACAGCCT	427
PPERSA_00002080	CAAATCATAAAGCAAATCATAAAG	TGGAGAGAAAGAGAGGTTTTTA	436
PPERSA_00035610	GGATGAATTTTATAAAGAATAAG	AGCAGACAAATAGAAGAAAC	417
PPERSA_00117390	GTATTAACTTCTAACAATTCTA	AAAGTTTAGAATTAAGCCCA	373
PPERSA_00079580	TTAACTGTTTCTCCAAATCCTTG	ATTTTTTCGACTGCTAATCT	419
PPERSA_00031570	TGATCTGTATGAAAACTTGAGTC	AGTATAAAGGAAAAGGCA	280
PPERSA_00130810	CTTGAGCCATTTTTAAAAGC	GAATTTTGAGGTGTCTTTGTAGT	373
PPERSA_00076120	TTTAAAGGAAATTAATGAGA	TTTAATAAGCCAAAGACCCA	328
PPERSA_00055830	ATTCCTGTGTGTGAATAGTTAAG	TGATTATGTAATATAGAAGATTG	463
PPERSA_00113410	GTGTTTTTCTTGTTTTTTGAAAG	CAACAGCTTTTTTAGGTTT	422
PPERSA_00109590	CCGGAATTCAAATCAAATAC	ATTTCCGACTCCCATTTTT	441
PPERSA_00073400	TCTAGCTACTTCTAAAACGT	AATAATACTTTTTAATGTCC	395
PPERSA_00069080	TCGCGAAACCATAAGAAATT	TTGTATTGTACATACTGGGTTAG	487
PPERSA_00041150	TCGTTTATATTAGATGGAGG	GGAAACACAGTAACTTTTGGAAT	311
PPERSA_00069770	AGGGGACACTATAATGATTTAAG	TGCTAATTTAACTGGTTATTG	385
PPERSA_00059730	CAGGTTTAACAACTATTTCTCTC	CATTTGGGTACTTGTGTTTA	399
PPERSA_00059750	ATTTACCAGAGTTTTCACAG	AGCTAGAAGAATGAGGTAAAATC	392

Protein sequences of 54 HGT genes:

>PPERSA_00089970

MKFNRNLNLKQTKFISAVKSQFDINQAQTPLSGRSKKSYHGNEFMGILRQKISSPNLIMRDAVLDVSQNSQQIPIFSFAGSGELESDGANNAFKEEQLKKELEIGIRQKKKKKVYSSLQEYYTQFYF
PNEFRLKQQYFSNTKFHEKTDLFYKPFEEVIKDYLEVFATCFSDVPKCDIRVQIIQQKETTEYIYQQFLNYIKDDIEEEDFKKIRHNFEQIKKNHIHQPIKYFEKLNGYQEVQLLASKILTTIPKDIFQKED
YHTVKNNPQIMHFLIQIFRQNYDKAKFILDVFIQSNQAITYLDYSLIKFQTWKHFFLKDKQNVQQQQLEIKDVQLILQHIENFRIFMFQQNINAIKEQQDQIVQCLTKVKILKSDQYFGKFPIQLIQ
QFLEDFISTFMLNRQYQNILTFDDTLLNELKQAVLSCLPPNIENPYIQKKDIISKIYIFKLENNLIQDTDVAQDDIAAIVYLALNKNVKIQAILVTGSGEAYCDQGIENIEKMLNFINYEDIPVLCGDNYP
LQGYFSFPKIVRDFANTDKFTFFTEQNQDQKQKQYKRQNIRKNQHAADFQVEFLNNQKEKNIDILVLGPITNLALSLQKDPQIVDKINKVVFMGGSLDVEGNLNLPNIHQSENKYAEWNVFSDS
LAAKQVIENYNLDFYLISLDGTSQVPVTKDYIDQIHNLISEIIIKNQNQESLQFASFFTNLFRKQIYKYPQSKLDFWDVLAAQALIDPNVCQFQKEKVQREENDVERQFRVYNNWFEVLNRKSQREI
NNGQIFRSDKGALNNSVEIEAVFITGSGEAYCDQGINNIVKTLDFLNYKDIPIVCGQNYPIQGYLQFPQIIRDRANSDPFPFLPLSNKRNVIRNQSAIDFQIQFLQNLEQDEKINLLILGPMTNLALSL
EKEPEIYKKLDQIYFMGGAVDAPGNLHIPGIYETENIFTEYNIFADSKSAQMVFANKNLKLKMIGLDGTNQLPITAEYLQNLLQKIEEKKKEFSNSLQLLNALKYGEFFVEIFYDWVRKYQKAELYFW
DVLAATAIIHDDVCTYEEENLYVQVTEISKEEENELERKFRIDQNWFPYHNRTSLREIDNGRILKSQELGNPIKYCKNVDKKIFDEAIENSLLGLEYIIQLQEKIE*

>PPERSA_00083530

MQKNTNFLQKISQYLKYPLIQAPMAGGSITNEIITEVTKQGCLGSLATGLMKPEQVYNQVKQLNKLMNNVPYNINLFVPEFQNKANRQQIENVWKILKQYDNSLGDLEGRKIPLQYQTDFGEIL
DIVVKNKNKIVSFTFGCLEGSEIERLKRKNEAFIMGTATNLKEAIYLQDQGVDAIILQGSEAGGHRGSFLFNKGENQQNKPELIQLEDLVGEIIPIIASGGISNKNQIQNYMAQGVDGVSIGTMFLTT
KESGIKAKNKNILLENKNIGTTISRCYSGKYARSLNSSFTKNMEKHLDELLPYNIQLKLTQYANTQSDREYGAIYAGSKYYDCQDITVKDLLQELLKGFE*

>PPERSA_00084980

MKKNIQLQPIVIPMNNEQDEYNLNENHVNKFNDESPNDSGTQNNDNNKKSSENFTNQQNKSESSLIDKNHYILLNKQQNQNPSKCKTINFIESEQKKPKHENIFVAKPLNEKQIAYFKKNKTM
HLSVQNVVNNYSSSERNEKMEKSEQIDNSSTNSYCSNSYKLKPKIFYNKSIKLQKARPGTPRYQGTNYGFDKKKIKFRHIGYDTPFQPVYYADNFDEPDQGPFQQDQNLNFNHSQNKNWVIIHG
LEQPKLIKAVCKQFVDVEDDLSHEQKEEMWNGWVADFVETHSNPTLNDIDSNQLYMVIKYLYFVKKNDSISIVVKHFSFVAGQNYLLTFLEDGKQEIDGEIINTTIDFEHYMEEIMNKIYKNDKKY
CKSNVYFILFKLIDKIVECYQNTLRQFSAQVIDPLDNEVIENPTSKTLKRLHNARTEMLEIKYATKPMQNMIKRILKRQSFYNKDLQNNIPGFDLNLQNLQSQSQMGTLQENQIDNGLIKSNELKN
QFKKKPSMQNKNQLQQQNSDSNGIIQSKTNKKSTYYNTQNENNDDSSIADSPMKQKNKW*

>PPERSA_00041150

MESNKVLVIYNQKCSKCRETQQLLEQKKEKNSSLSIDYHYYLQDGISEQLLEKIVDLLKVSDVKNIMRDTDQLYKDLKIKESNFSQQELKNLIIKNPALLQRPIVIYQDKAVIARPPSNINEIL*

>PPERSA_00117390

MIAYAGGEVGNTTEQPLKSGASWMTILTGNWANYHKVVSNQNGPSKTSKTIFNYIKKQKPHSKLSYIYHWAQFQTLVRNDLNYIDYHKKLDKGKQESYIYDLFLQSYQDDMIIPDFTFIQFDNID
HVGHSEGLSQNYNDQVFETDKIIGKILENIEEIKAQNQNLEFLIFIVTDHGRDKKGKAHGNYIIQQKTEKQSFIGTLQPELLNDEFHKVIQAPISDYDNIYSYPSIANLVPTILDFLNIEKGQNFNGISM

IGKLGLQKLMKIDNKYYWAKIYNPNEGQKISIYGDGKLIDTIENTVSQWPDSNFQQDLIKQYTKITFVYNQQQVTYYNKNYSIPFYYFYLNNIDFNDTYFLIFFYGLMISILFNVFVCFALSVKKIIIKM
FVKKKSKIPKIKDDIKSENDQSQQIYQQMIDISIK*
>PPERSA_00031570
MNKLQKKLRDFFVRITGGVDNYKGKGIKQIHQNLEIKISEYEAFVKIIYQCLKNAGFKKEVVDQFLQKLAEYKEDIVFDYNVESTVDSSFHTDQDDVVFINH*
>PPERSA_00073390
MKQISNTLLKTKSLNKSQQYTFAYGKIKEDLSKQLQQLEVDGLYKRERIIDSPQGTKITLQDGSKVINFCSNNYLGLSNNQRVIDGAKNAMDKYGYGLSSVRFICGTQSLHKQTEKKVSDFLGTEDTI
LYAACFDANGGVFEPLLTEQDAIISDELNHASIIDGVRLCKSKRYRYKHSNMQELEQNLIDAQNQRYRIVCTDGVFSMDGDVAKLQEIADLCDKYDALLMVDDSHATGYLGATGRGTHELCGVKD
RVDIITTTFGKALGGALGGCTSGRREIIDMLRQKSRPYLFSNTLAPAICGGTQAVIDMLSESTELKEKTVENARYFREKMEAKGFDLVKGNTAIVPVMVYDEPLAVKMANEMLKEGIYVIGFCYPV
VPRGKARIRTQLSAAHTKDELDQAVEAFTKVGKNLKLI*
>PPERSA_00098990
MSEKMIKLEKKYQNQPEFKLCTAKTANTLSRPSSEDSNNSNENNKNHDIFGFEKDIQQIAIDVTKGAFTQTESVPVVNKNKYNFNLKNIIIIVLILVSIYLSTIIADLSHYKKQKQLNSHKFDDVDFSLN
ISYEDFYEQTKLKPLSQLQAKCSHNSFEAGNYYEQLNFNKNTPYQGGSLMLEFDIMHDIQENSEEYGYYMSHLQESHDGQTLLDGLQQVQDFHNQNQDHLPIFLTINIKPQLRNLEYGFNPEKF
YYGLERTIVSALNINDIYTPAELLNGEKNLFEAVTNNGYPQVQQLLGKIFIVLDVSQEDNNDPLFYQFYENYINQVEQSLMWGTLDTKLIQRTYYTSIKQYMEQTQNYNKIFINMKANYFYDSDKY
AYLKGIDVLKEAQNLNLLTRGYVLNNMNNYIKYRDMGMNFLCTDKIFNHNFAVAY*
>PPERSA_00045220
MDIVQIGSARADPVKFMDLVDSSQTKRCQRYVEKCNGPTELFINKQKKSALDYLSEAFKGMEQYKDKIGMIIDSSTTRGFTEPSQCSLYAMHLGIRYAACFDVVEACNGWVRAAETAYYYLNCRE
ELEDKYVLIINNEYPPETRAVVPKSPDDYQACASYYVGLTFSTVCTVTLLKKSNNRSWRFFNSSDTIYASHINIPLPGLAKDFCPTSKEYSVYFTECYGQFCLPRFHAMEEKGLEVLAYVEKRKEFFED
ANVICHTYTKKVWEELFQFNKFKKLSLYFNETGNLGSCSFPYIAKDQYGTTLPKGEHIAFMGTAAGGSGSIVTFVHEQDDGPLDSQYPKVKKVEPIGRKLLLLKLGFRALFKKILCQKKKRNYKSYSP
QGAVDQKTE*
>PPERSA_00055830
MSANNKSWLYLPQNIQEKTQNSQQKPSVCIIGAGIVGLTSAYFLQKSNFFGKITIIEKEKEPGTAASFVNAGYLVFSKVIPVCEQLRNINLSIFKYMDINLENLYQATRFGIYAYYDFFKGALSSKYKQE
RTETYSVYGERCKETFWKILQDEGIKESEIGLQRGCNYLYRDENKFKNSLEILEQQKKPYIIQNIPLKQNKNGYLQKMITNNEDCTASSWHFLNILLKKMEKKGNFQLITGKEVTELIKNEKNNRIIDG
IKCKDGEIIKSNYFIFCNNGLIQHIISYPLVIMSGFSWTFYKNNTKNQALINEVDNCPSVVDYEKCIYYSIYNDRLRVTYGFYLNPNFQKLKNDFKQYTPNFPFWKDEAEKQVENMTIGTRCVSPNG
APFLGQLGDYENAFINTGHGFIGWTLSTYTGLLLSKIILQKMGHQIEFDQIEKNIMKITNPKRYII*
>PPERSA_00035610
MSTEQITIEQQETLIKRQQNPHKNQDKNLHKNQNKNQNTNTSPTQQQNIQQSHKKKIYHKHVKLIHKAYKFLQSKVESPEIKKQFLSRQIEETPKYRGQIHNYGKWISLIPSILLIICARSFKARRN

AIIYSIGVFWMQFISSKMHCRPYQTAPTWIRKLDNISIFLYIGMNWFTIFEQVMIEDPKWEVTFGLVTVLMVVAIILRALTLHSPRWLISGIALLMGWLSIILLPTIIQKLNTYEQTVFALGGLSITLGA
VCWALQFPKKKKGSHWTPHETFHLGTFYEEKI*
>PPERSA_00107980
MAQFLEQQVEPDAIMKFFPFFYGYIALLMGTIGKIMSAPGQSVCIGVVIGPVQIASGYSNTLISILYFFATTLGGFSLPYTGKYIQKYGQRKSISFISVMLGIACYIFANVHTWWSLFIGFYLLRFIGQG
AIMAVSVTQVNYWWVTLRGKVMGYASAGLSAFLTGFAPLLVNIYVGEYGWRATYELLGNISFFVMLPLGFLFFREPPEYYGLLPDGKKNNALIEKPNRRTINEMDKEMENLLVNKNEYQISNNQ
EIIQEDIQDLGKQNKKKRFSIVIEQTNEEVHWDVKDMLKTWAFWTSCVSLFVASLTSTAYFFYMNRIFKENGIPDSTVNYVYTFIAIISVIIPLSAGQLMDKVEPHNMYKVSLLGSFLAQLTIIFINKD
SFFLVYFSTFCEGICFGIAIIVGNTIFANYFGRKNLSQIQTIAACVEIFGSATGPVPYSIIKDIFDDFTYAFIFGQILCLLAIYLSHVKGGKPVNPKQAQVDIDQEQQLSSINLDK*
>PPERSA_00109590
MDRVGGRVFTTETGVDLGASWVHQWQHKDNTVRKLIEKLNVPVVGENSDQEVLEDFFDQVSQTRVDPAIIMKAQKHLDRIIEISIQKASKLKRDVSIYDCVKEELQQIDNMEDELYKRVFYQFL
AQCLNNEAATLKELSAKMGVGNPQTNENQKCSVRETQDNYSYKDYEDKIIQQGYGNLFQDLSQNLNIKLNSEVQEINYIKNQTMPFNFQIKVYNNKTQQTEFFYAKYLLVTCSIKVLQNDVISFQ
PELSQQKQKAIKKLGLGQANKVVLEFEKCFWDERIYSVCLAQQKENFADFPIFYNMKKYKNKNILMALLSDDQAIISERKTDQELVDSAISKLQQVYQKNYPEFDDSYFKVKNYYVTRWGANKFI
QGAYAYYPVGANHEDSNELLVPEYDNNLYFAGDATIADLIGTANASSYSGEQQAEHIIQKYMFDQSQEVLIPKL*
>PPERSA_00029910
MSYDIYAKSLSQNIISLTANSTDPLFPIENSYDNNIFTEYKSNDESVELEITYDIQFIPIGVRIVSGQSMMNAPLYFHLYAFNTYTNDWDTLSYLKFVGDLDIYFQYLIEIDDLSNKKSSWGGQYVQISEI
SIIKPTSISSQYYEQVNRLAEDAFLDFNFKKETFSWLNYEDTFEYTIKTEIYYQQNLQSTIDALPTGLIFNQSNLQISGTLNPQYKHKILKIFITAKDSSNTQQTEQFEIDYASIIIGYYIFEQENLIQNIASN
SKQESGSLNSVIYDNIDKSYQFTGAENQYIDTNFNEFPAKFSFFMKIQTTTTENCILATNRQCGTTCTDEPGFTIYMQSGFWSVIVVDENQNFVTVEYVDIINDGLEHVIGFYTILSDSLVTYQDGFE
LFAQPNCPENTIKDCTGRCVSDYYYERDKCITGLFDAFYSFEIPYLECPELGCKDNCYECLPYNPENKIKSINSLKIGDFFQSEPSYYSGKVFFIKIYNRELTFQEVLGIYRQQNCYQWETPLQETYNTLY
NGILRTKTLVSVPTGNIISNCQYLYEEESVLDPSLLTYELYVNTINEKNVYIDNHILYLFAEEFYFGQEITIYVEGLYDYQYTSMFEPITAKIKGKYDEMYEIVAVVSGEQISHYNVINIYDIHKGQINGVTYI
FTTKLWSQIQFEKIEYVFNKINFQQAYYESLSYPQEIMSSSRLYQIQNTYFLIVTVSTNDLDGIKIYNVTNPSDPKVVGQISEGIVTGFEAKIEVVQIDQVWYSIFKTNSQIFLLNINDPTNPYNYSGFS
VYSNSIFSFQINGLQYVVLSILNQGVQILRISDASNGQFQSVLNEIIFDSETNQSQSVTQSKIFAQYDAQNSAVVYYLTIVLNEVGFQTYLLDVSDTNFFTMTLLYQKYLYGINDLQIFIVGNIYYLALM
GSDYGIDIYDTRDTKSPVFIQHFDYKGVKAIEFYENEYNKYLIIAQTSKEGVSAIKLDLIYNNTQTNPYQTLLSQNNYENEISDMTTKYFTQAVVYYNYNNQDYIASIIQKYIANQMAYTFYLQIQKVT
SFYQQTQVLKLQFQWLQTRFSSTQSKETILQLERAGSNYIIIYRYYAYTRLSIFDITDLDNVYLVSDVNGEKYMQSSGGLAKIYKINDIDFVFCYDLDQLLMYDISDLSNVIIKQRFTGIYDFQYVQAID
FYQDWSGQHMVATSMYQTSFWKIKYDSNFDYYYGLPLVGITWLETTVDLKRYDNWYNFGYVERSFTGSASIYNNRYIYVVSNQNGIYIYDYTDIQNITLLKYLDFQYATNTQFNYMGFIKINLKTY
LQVCAQDSGLIMIDVTDPENSGFLYSFETNLAMQFQTIIKDNEYYNIIADQIGGIRISKIQQYGAYPIISQEIENGSKQFNIQLKIYQPSYFQPYFTSGMIKLLDIQVLKQNSQLNTFTTIPSWMTVNL
DQQIVSLKPSSKAELDEINKIYYVYSLKIDEQELQEYVEAAYPTSSLDYEKLKLELISYGHITKKMFIQEYIDENYNLQLSSQFDDYLEGITGFLKTQQFYGHMLANTDYITASNNPPVITCSEQYLKYEL
QSKQDTLCPQNQIQEQLNSQLQISLGLKVAKVGKYISFRFSENTFYDYDEEKLTYSISNIKRTFENKTIIDGLYYTDISWLAFSDEYRILQGTPTTEYYNNILEISITASDGYDNTTALLIIDHSTIPPKQNK

NVDNLQKQFNSQNPNPQIGQQILFNFQNSIPFIDDDKDDLKYLAYKFIKSQSKFIYILDLNKYDTANYLSFNNYTLTFQGTVPKNYNKETEIYCVQAFDGYEYSDCQIFKIEYNDKAPKLKSKIGNQK
VTVNQNFEFTLQSDSFEDEDKQLSITATQSDGSPLPDWLQFDSSSNTFYGTPDEIVNVEIQLTATDIEGKSVQTTFIIDVQYSIYYLGQQLQMYGGILLGFIGLFGVWNYRDEFHNLFSKSRKDCLVI
GETQYQKYIPIISQEFQNTCEKAWNIFTNNAKNNNLRTSATFDIQNLIINNNFDIEPIFQAMKSILVDPKNKLDPKQVDKIFQETYNGKYSKDKLKTNFGIIIQHLLNHFRLQQDGETNQIYNQIKKK
VQNQIITYASQKLKSPLDWYKEFVEILEKDLDEIEQNKFPNLSVKKLEIFDYVMEILDPDNKIDENNYQQDYLYFYNSKFVNNKKNNINKDKTTQDQQSVQIQSKKNKEINKNNQKATQQHVKINF
KLLKDAILSEAQMQETIKVAKSRYINVVKALRVRKHKFFQLSLIRFLKKEQYNIEGHKNDPLPSWLKYQFLQKTAILLEGIPQSTDDLELILQVQDSKDFILREYLLEIKKPDSQKKLKLSKSFTNHPLLYS
KANQNKNQQSTAIFDNYNYPKQDTNQQSAQQAILSNQLDSFTNTTIEQLDEEGIKTRASLINRNSIYKSKLYKSKTKVQENGDKNSQKTNIIQNQSSLLSLSQKSPDQKIGKQQSIKINLQNNYLY
QDEKSDTMSPQGCIFSQNQTLFESNDKLVNFKNENNDAINNISNSPNNQLSKKSIYAKFQNQIINSSSGDNDSASQNRDQDLQQNSNRNLISPVKVKRQRSIFAEFRNINNSPDTDIQIDISSNN
SSNSNNNNNNNNNSNCNQNNNDEVNNKKQKSDNQISNPSKSLKKTNLAYPKVHEQISNDDQQIEIDDDEN*
>PPERSA_00073400
MSQVPKYLIIGGGGQIGPVLLKQLRNIVGNQNVVLSDMRNDLDYDLMNSGPFYQLDVTNGEALSEIVKKEKITGIYHLAALLSGRCEEVPELAFKINQGGLQNVLEVARLNNCQVFYPSTIGVFG
KFFNPNNTIQDSHLRPTSIYGITKVFGELLCDYYHNKYGVDTRGVRYPGIISTNILGGGTTDYAVYMPQHAVQKKDYDCFLLPDTRLDMMHIDDAVNATIQLMHADPSKLVHRNSFNITAMNFTP
HELHKEIQKYYPEFNVSYNNINNLKQKIADSWPNNMSSSCAEAEWGFKPIYDFESMVKMVIDQLKQEQQLQQSTQSTQQTKENEVNL*
>PPERSA_00069080
MSQKISAITGAFSYTGRYVTQILQQQGVQVRNLTNHPSRPVPKNQINKDQIDVYPLNYDDVNSLSKSLEGADTLYCTYWVRFNDYKGTTREQGIQRAKNIIQAAKNVGIQKIIYTSHTQSSLESPF
AYINGKAQMEEYIKESGISYGFAKPCMIFGDTPQESIVANNCAYLLRKLPIFLIPGQGNYPVQPVHVRDMAQLMVKMAEDKNQNQDFDAVGPEIFTYNEFIQLIQDATNSKVLKIKGLNTDVVHK
MITPVEYLLKDKFIEKDDLDLLTNNYLHSKQPPTGSIKFSEWVYQNREQLGKRYINSTDLYYTGETTIFDKVQKI*
>PPERSA_00035440
MTIFQLNKINNETLKSQVSINTKNKNKQKQVLNFQAGSEKSIQSQEDTSDDSFKQLDFENEQTLDEYYNSQLDLELTQVTAKCSHNSYEIGQVQEQLQFNETHPYQGGSQMLEFDIFPQNITDEA
LNNDTQEQQQYIFYTFHDQPKSLEECQTLSNFLDDIKEYHTNNKNHLPIFVTLNIKQWITEQYTLDAKIFYKNLEQTILNAFSIDDLYIPDDLLNGEQNLFTSVQKYGFPKVSALLGKIVFLVDCAQG
GNETPKYTEFIQYYLEQDISRNLMFNTFDSRLIQSGQFDSIYDYMINTGNYNQIFINIKQNYRKDLFKQKYIKQFSILKEAQQLNLVTRGWNLSDIFLYTVFRKMGVNFACTDYIFDTEFSIAY*
>PPERSA_00076120
MEQTSDKKINSPIIKGVQYYPNFIENPKELFEFLDKNCNWDTSMKARKTVSFGVPYNYGQMSYENNNFPKQLQEINEKIEKKIGFLPNNCLINYYEDGLSRMGYHSDRTDNLEPNTGVVIISLGET
RLLKYRKIPQNNNDNNDNQNENENYDKNWENKNEKNQNQEFWNYYLEDGSLAYQSLEIQKTHQHSVPMDKGIKNPRMSLTFRRVIQNKN*
>PPERSA_00069770
MLEYSPKGTVPVMVIKNQNLENNKFQKQEKNQEKVLEQSLDIMEYILSLQDYNQILKKYQNSIEQQNLIKKNDDQFKLLLDKYKYWTKYPGNNQEEYKNDCFIFLKELEQLLQKNNFLFGQDFSF
SDIAIFPFISGKFEVQLIQRKNCSLKQFLNKETKVLYKKKQRQFTIDVQVAKNIFNQQQQIQIFKEFQNQNTLSSNSTQDVGSSDRVNQSPQIIIVSPNDQNSQYQQQPSILETKNKTLSLNQRYYQ

QNFNYLTVPPFFRNLLSDKISKNKQKLGNKDLKIQISHLSNLKLPESNINKEIQVDYCLSTSKYILNDRSGFRMEYSVTKKDNEYKNQNDFYINHSQVLDIGQKYLQKAILKEDMTLVIRVSQNIQEY
DQINNINIGWCKVNLQNFMEKKTLFEGKIKSQLWKPPMSLQQNQNENNNNNQPKICQQNQIIILSLKYI*
>PPERSA_00054440
MTKLQSNLSQNNLCIVQSYQKQQYDNQQNVEIQQLENEKNQTAQVLQKNDKTILISYRNLQSVKDKLQTSYSDQQLQSDELLIKYQNIYKQNDQLQQKNQELKQQNDKLYEKYNNLDMKYRQ
LYYGEKIDISTYDGLYNQNSNTFIEYYNSQLNLELTQITAKCSHNSFDIGRIQDQLTFNETHSYQGGSQMLEFDIFPQNNTDELFNPQQEYIFYTFHGIPKSLDECLTFNGQLQDIATYHENNSQHLPI
FVTLNVKQWIIEEYTLDTQTFYESLEQSILNVFDQQDLYIPNNLLNGESNLFSSIQKNGFPIVSELLGKIIFIVDSSYQQDEKAEFTKFIQYYLEYGLETKLMFNTLDTRIIQNGKYDSIQEFMNETNNY
NLIFINIKQNYKKDLYPEQYKENGPQFCLY*
>PPERSA_00079580
MKYFIIQIITLFAIFNFSEQVFQQNDLIQENDKQNQHNQQKKVLLLGLDGLDYRQLSNGITNGELIGLSQFTYQKSYCGGEIGTPTEQPVKSGPSWMTILTGNWMNQHHVSTNLSGPTQETKSIY
RYIKQANPGSNLAIVSHWSVIENKLLRNDRQDIDIIQQILSDENDNNIYEKWLRTLKNGIPDFTFFHFNDIDHSAHLQGFGETVNTKIINADKVIQKIMLKIKELISQNQNLDFLVMLVSDHGRDKY
GWTHGNTIKQYYSEKLTWIGTNQPQIFNDEFFLPSTAPIQKLNSIYNFPPITNIVPTILNYLEIDYKKENINGISMIGDIGQ*
>PPERSA_00125500
MQQKSEEQLNQEKKIRQQKPIQLYEYNPIWPKLAEQSAKELKEIMMPDLDNDPKIKGFQHIGSTSIKGMAGTRFIDLAVMVKDVPLSNEVSQNLKKNGYLDLGMDHMDGGQWFQKRNEDQ
DFDKVGEAVVIHAFAGEKGLQSFKQFIAFRDYLNHDEKAKNQYLFVKKSLEKENQPFIGYKKKKSEFVKQISEESVKWADKNNLWEKYK*
>PPERSA_00057430
MKIKVFEQSMYDGTFETINLNDEKIQEYYTGEFQDIQIASCQDCVVQIYLVFERQDVIKQKIDFGQIFYPVNHDANKNLLIGYQQNYTVKRIYFPQTISFVQMYQLESDQYELEELNINYNNLLLFPT
EDIKNSQIPFKAGGEYFVVNFYQRDLNTDYIYLLYYNKESYEFEYKIEYDYNVQIYENSDKFLIQYTSTSYIFYYKAKKGSNVINFNNVFENSYVYINKCKNGIQDCNFPDYLQYDKQIDNTVQTNNHR
FNLTERASSNDFYSVFIYTYYIFDGTEQSLETYSSTNSKKSYAPNIRLVLSDKYIYHSDTPYWVFVNCITQPNKGSDTDASLEPTKKITQSYSGTDYQMKLIYNSKGKYLSQAASNLGVQTADLAGILKV
ESSGNGFTASTGLMIIRFENHWFYKLYTNNGQDSAKVATFNKYFKFDSTSTWKNHYYRKSTSSDWISFHGNQNLEWDCLQFAQSLDDTAAIQSASYGIAQIMGFNYKTLGFSTPQKMFLDMSG
SIKAQLDGMFNFIKNSSKCYNALKSNPTDYYNFAYCYNGSGQAATYGNLIKDASASYKKITAAIGEDDNNSGPVLQMAAGDVIQFSSQINIRNGPGTSNTVVGTTSTGQEAVITSGPSSNNGYSW
YKITMVPNGVQGYVAHSSGWFSPKYQIRQNDTVKTTTSVYLRSTPGGSSLGLLSSGTQGKVTGNPVVSGGYVWYPIKVTSSGSQGFCAMGSGYLTKI*
>PPERSA_00050920
MDIVEIGATQGQNYNFMDQVHPSMKEKCEKYVKKSKGPTNLFRNLDGRTPMQFVEDAFKPLQKYKEKIAVVIDGSTTRGFQMPSQASLYSQHLGLKPNQYFDMVEACNGFCRALETAYMMF
QTQPDLEDKYILIINNEQPPDTRGVLPESVANYKQVASYYVGLTFSHVCTCTILEKSDNYSWKFNHYTKTTVATNINIPLPYISSLFNPKCDEFDIEITDCYGQFCIPSHHDLEKKAFFSLNIVENDIEFY
KKAHVITHTFSRYVYERFFANSHFQKLSVYYNEAGNLGSASLPWILYDNYGGKGIPQDQNISFIGNAAGGSNLTLQFSHKSKKYDCPQVVDDRHKYNKNLLLLKIGLRSKFYEWVLRQGKGTPIST
YDPKKNQDNNKNQVIQTKKAWEERVKQMEQHLEENKWYPVDNQLQQFKKSNKISSN*

>PPERSA_00098980
MQQKIHPSQDISLAQNKKAYINQNNYYNKATRDIDIEPSLNQDKKFQKTNTNSTGSTDDSGGETYDIEKNDFEKQKQKDENHQENAQFVTQNAPYYLLPLKNFTRFPQYENYYEFMKNQNLSR
IQTKCSHNSYDIENYYEQLTFDNKKPYQGGVLMLEFDIYPKININSTIQQKYKMGLAHTKDDKEEKPLINALQEVNEYLQDNPQSLPIFITLNIKEWLLDLIESPKVFFDSIEFEFLEIFSQNQIFTPKQI
LQGEKNLYDAIIKFGQPKVSQMLGKIVIIIDPAQSNLKNQKEFQEIIDYYTNDLSDKLMFATLDSDLIQNGDYINIKDFMETNNKYNQIFVNLKGDYTFQDKKEKYFKTFDILNEAKELNFISRLYNLN
QIDQYILFRDMGMNFLCTDHIFDYPPYVAY*
>PPERSA_00036040
MNINGIAHIILSVNNVQKSKLFYEPLLTYMGMKKVKDEKQFLYFVGGRTAIGLEQRNILTNEDYQITENQQNVGLHHFCLRAKSREDVDKAYQKVKTLPNANIVQSPIEKQWAPGYYYTLFEDPD
GIRIEVNFVPGKGLLETKKKVEFNAKL*
>PPERSA_00047700
MLKGILSLILINIIDSYINHKLKNSHDKYFKYITLKRYLSFIIFPLLILEGLYINKKIPRLGVPADETKGLIKNSNSENPSENKLFKILYIGESTIAGVGLNSHQEGLSRQTALKLVQRKDFKGIDIEYDSIGQNG
ANIKYLLEKILPRFDAKNFDTVIIAIGVNDIIEQTNLKTLKKQLLEVIQKIKQQNNDIKIHISSPPPMDKFKALPQPLRSFLGYRAIQISHIYRSISKQQENVFYSLAEFEYDRKFFAPDHFHPSSHGNFQ
WAEIMAQSIEPVIIHKD*
>PPERSA_00021250
MAENDIVGLSFGYGQDGEVQWLQGFGHANINENIMVDAESTEFRWASMSKQVTSILSYKLNERGKLDLNADISKYWQKYQRPNYFVIKCNGQDFVEYDDYKYQCAANDFAYVEVEQDTIITQ
SMLLSHYAGIESYGDGTTNPVPPDSKKNDPRINDGIEWAMQYFMNAPFVSQPNSSYAYTTFGLNMAGVVCTYADAEYQGRYEDLARALVFERVYGNVGSIQPDFYWLEKYRPQRATGYKNPQ
QLSVDNDVSYKTPGGGYISNVKDALTFALNYRNETLLTNDQKSRAWTPYNEKINAYYGMGFQLWYQQDQLLSVGHDGSQDGARSMMRYYPQVDVAMVFMSNSENLNTSKLLKGIEKILWR
NGVPLPENWNFQEDTEQVQFQRDIQI*
>PPERSA_00131510
MDIVEISAVTGQQVNYLEQIPENFKSGCKRYVDKCGGPEFIYKEKQNKTPLQYVQEAFKPLEKYKDKILMVIDSSTTRGFCEPSQASLYAQSLGLRNSQCFDISEACNGFSRSLQIAYQFFETDINFEE
KYILIINNEQPPEGRAKLPESDRNYKQIASYYVGSTFSNLCCLTLLQKSENYSWKFMNDSDTIYASHINISLPNLGQGYLPIHELYSVELNDQYGQFCIPKLHQLEEKAVKVLKYISKRNKFYEQANVIC
HTLAKNSWQQMFQFQKVQQLSLTYNETGNLASASLPFILFQQYGKNLPKGQKIAFMGVGAGGSGVVIEWEHQKTNENVKNSSNNNRNHKYLTDKSDSSYKKWDKYTLLLRIFLQETKRKIFC
QKRNKNKIKNGYNKEE*
>PPERSA_00059750
MFAAIICFGIGLTTVTLLGQTMARAELLLSFIGVIFMFIALLSKDIATLKPSDEQFALLVIGSFLSGSAIANFQLIVEMVMWYPLEIAGTVQGIFAGIGNLFLGAFGLILINCLQNGLSLIGGYIIVLVLFVIT
LPLTYIYLQKTPLTQLIDQKMEENKATIIAEFCGQQNFPSKFHIIKSNYLKGYIMAYHKTTVYEALFQNFTFSCITSFTRVFTGYLTDKITGFLSTQISLIIIIISSLILSFSQSNSPHSFMHIGGLLFLAIGIGF
MMAGNYKWIVEIRYNHILAIGMLAGFFGALGGPILYLLLSYFLQLQEQLFQQQIKY*

>PPERSA_00113410
MPKKVVIVGGVAGGASCAARLRRLSEQVQITMFEKGEYISFANCGLPYYIGGVIEDRSKLLVQTVDKMSKRFNLDIRNYSEVIKIDRQNKQVQVKNWKTNEVYYEKYDKLVLAPGADPIKPPIPGI
NAENIYTLRNIPDTDRIKKYIDENKPKKAVVIGGGFIGLEMIEMMALRGIQTTLVEMADHVMGPLDYEMASLVHQHLKLQGVEVFLQDGVTEFEEQQDKSIKMKLQSGKIIETDFIVLSIGVKPEI
KLAKEAGIKCNKGIVTNLRMETSDSDIYALGDAAEVQNIVSKQRALIPLAGPANKQGRIVADQITGRKVSKFKGSQGTGIVKIFDIHAATTGLNERQLRQLDREYMESITHSSSHAGYYPGGFVTAIK
ILFCPYSGQLFGAQVVGAQMVDKRIDVLATAVRQKMTVYDLEELELAYAPPFSSAKDPVNYAGFVGANMLKGDVKMIQWNEIQSAVQNEGAILIDVRTPEEYEQGTIPGSINIELDTIRDNLDKF
PKDKKLIIFCQAGLRAYIASRILYGNGYKEVYDLAGGFKTWHYAFLKQSNEGIFSRDKITSQGMVVQEVVDKALNSNYVYWDKDFENFSPIKAKQKLESHPLYDANIQKQSQQN*
>PPERSA_00130810
MQSLKKVLNNQVAKSQFKNLYQDQVVYAFASKYSLKAIDLNDADAPQRFVQSLKDTGFGVIKNHPIKQERVQSIYDNWYKFFQSERKHEYLYNKGTQDGYFPPNVSEIAKGNNVKDIKEYFHIYP
FGQIPPELKEEAMAYYKDTSKFASQLLQWIQDFTPEEVKKNYKCPLPEMIRDTDQTLLRVLHYPPFSGKEEPGAIRAAAHEDINLITILPSANEPGLQVKTLDGEWIDVPSDFGNLIVNIGDMLQEA
SGHYFPSTPHRVINPTGEATKKSRISLPLFLHPRPDVVLSDKYTAESYLKERLIELGVLY*
>PPERSA_00011350
MSIGIQSQTQQQLEYYYHLKQIIQAQPNLQKIKNIKEKLQQVKKNILPQQSLQLLELQQLNQNLQQQNQQQQIQQQQMQHQHFPFSVKEICEQHNIELLVQKTMNSKKSIQFLEDADLIVLGG
VPVLKEIIFKRPKYGCVNTHSGILPDMRGTCAFIWSIYYDIPLGCTSHYVIKEIDQGALINIEYLDVKRGDTLENLFWKQSDLCAVSAVNAVKKLLNKEQKFESLPNDLNRGRYCSIPGPEIVERARQIL
ENQEYSHYSSEK*
>PPERSA_00086300
MDIVAIGSSQGNEVDFMSLVDSSMKEKCLKYQTKCRGPYKVLRNDAKKTAVQFLEEAFKPLEQYKDKIGVVIDGSTTRGFTEPSTASLYAQHLGIDGPVCFDVTEACNGWARAVETAYMMFQTN
PNYEDKYILITNNEHPPMTRPMLPKDPSDYRQVASYYVGLTFSWACTCTLLKKSDNYSWQFIHHSDPVYASHISIPLPQIAKGFTPKSDVYEILLNEEYGQFCIPRLHQQEKKIQFVLGTVDQNEEFY
AKTNVICHTWTKYVYEEFFKNHKLGELSLYFGEIGNVGSCSMPLILHDQYKNNIPKGQHLCFCGSAAGGSDIMLNFVHGIETEKKLQKKEKKSNLYVIYARVAWRRLLTKLGCSRRQNKVKVQADT
KTQ*
>PPERSA_00032590
MKEKQNQIQNLELPQKQEININQQQQNQELNKELNKTYNTNLITQNIEKQQNQEQQQQEETNLIQSVLCVHGDGKGWNGLFWKNIMIPLAEKGFQVYAIDMPGCGQSQGNKYDFRNKGV
EVLDEVIKNLGLKDIILCGESVGGRTVINFVNFVKQQPKLQNNVKIKMLVLTHPVLPSTQQMQEYNNHKIPTLVTWAEDDIQHPYNGPHGVRYIKQMLKCEILSWKEEEQYSISEFYKTSYCQKFE
QLYNKNYKNVQINNKR*
>PPERSA_00043810
MEQEQNQEQKVIVCALYKFVRFPSFKQFRPQLLQFMENNNVTGSLLLANEGINGTVASDRQGIDNLLKFLNSNEILSPIEYKESTAFKMPFKKTKVKLKNEIVTMGVDGIDPLKVVGTYVEPKEW
NKIISDPECVVIDTRNDYEYKIGTFERAIDPNIHTFTQFPEYIQKNLDPKKNKKVAMFCTGGIRCEKSTAYMKQLGYENVYHLKGGILKYLEEVPKEQSLWKGECYVFDERISVNHDLEPGQTQPYYT

GRMENLKSSKEKKIQQNLQKNEQEQVEKEEQKQNIQQNDNNINNNEQQQQKQEQEQEQKQQDKQEIQPLQVQ*
>PPERSA_00002080
MYENIQDPKKKKQPQKQIEEPEKSQIYTLYINHDSLEENTQLLETALQDLHNFHQDHPKHLPIFLTINLKSYLKDYNINFPSLLQKIETQLKTSLSLQNLFLPKNLLKTHASLLTAVKNDNYPKIQQLLG
KFIVLLDPELFSQEDPNQQELLDFYNNYSQNVENSLMFPTLDGRLILVNFQTFAEFLEQNQVQNQIFFNMPGDLYESDYYYLQEEAQNIIKEAQKQNFITRVYTINDEEFYEYYKNQKVNFICTDA
VFYNLFAKPFF*
>PPERSA_00117970
MEAIDNQLTGNNFKQLLYTDRKINEQAQKVHGLKNEDLSDKKKFVEIIDELKEYLEGHDLIICHNVRFHIDMLNKAFKDAGGDFKIQKTHCTLQSSKKYKNKDNKKNQNSLVHLCAKYNIPYENKT
KSVLLVSKVVADLYFGQPEIFNANQEKEEQVEVEEDIDIYQILEEHEEQQRNQQLQLNVEQQSNNQDESFDIDEILQEIEMEKLELQNLKKESDTEEEISDIDEFLEQNEYEKKEKQNLQQGKNNN
QQGKNINIGEIQIKTYFAQNEQ*
>PPERSA_00086310
MDIVQIGVCHGEKINFMDQVHDSMRARCERYVQKSGGPYTLYREKNGKTPIQYLEEAFKPLQKYKNQIGMVIDGSTTRGFTEPSQASLYAQHLKLTNAICFDVVEACNGFSRALETAYMVFQTQ
KKFDDQYILIINNEQPPETRAVLPKDDSDYRLVASYYVGLTFSNVCTCTLIKKSNNNSWNFQHHTNTNTASHINIPLPKLAKGFLPHTDDYEVILNEQYGQYCLPRLHQVDTKIPFVFGHIEAMESYY
KEANLICHTFSKTVYETMFANHEIGKLSLYYDSCGNLGSCSLPFILHDQYGSVLPKNEKLAWLGNAAGGSDYMLTFQPWSNEEKKEYKVRGKYITWITLGYRVLKNKLLCSKRKNKKVGLKSQ*
>PPERSA_00056740
MNILVAIQIKAIFILSILQQTYAQTILTPQYNTSVPNLQEQFDAFFPNTKIYDTIYFPVNLKCFMDYNNSTQFSYSLLTSGSFLSMTYTSAINYLLFQGTSTLSFINTTHVITLRVSNAHGYSDFQFQIDYN
NVPPRVSDNYVSLQQEFDTQNPYPQIGKIISYKFIVNPFVDDDNDQMIYTAEYRILEPDGVTERQTWGQLTGTWYYFNPTDLSFQGKPTMVALNTYYEIRVKASDGFVESQYLHYTIELLNNKPR
LNTNNLSLQEQFDQQYPVPYNIINKKLFFVTIYSIFIDDDNDQMTNTITYIDDPAEVSLYNSGNVNSVTWLTLASDKWILVNEKTLYFTGLPTINEIDTVIVFKVVTSDGFEESDPTFLKIDFSDGAPVP
ASSLQSAGALQQMFDKQNPNPQIEREIKFQIESIPFTDPENDPLTYQVQYQDPLASDTTAWLDLLSDNWFVFNENTYSFKGTPPVEYMDTLYNIRIRGYDGYKYSVYLYFTVDLENLPPAIYPDVD
DLQTQFDSQVPNPQVEEEIYFQISQQPFYNQDSVDNLYWVVEYYDDDLGAYAELYDKWLEFNNATLVFQGTPSVDLINTNFKIKVTTNDGYKSSSSMYFTIDLSNYQPTLNLDIGTLQEQFDFLFE
NNQVIAHCKETFLFEYDSDTFSDKDDTSTESSLKYTAYIRKFSFQDQIWGQFIEITDNSDTWLYYNSKYSRFYGEPGVEDLASYEVKLVVSDGFDEIQNIFGFSVTNSPPYLNKNQNLQSQLTKNPV
VDSDFEFEFSSSTFIDEDKDDLDFTITMENGDMIPDYLIFDGSSRSLSGKFTEIRKVNPTGLSAYFYQEALTFVIQGEDNGEELVSDILVIEVSPSFWYIFILVIKFSSPIVGTLGLYKYRILIFELICKKRYQY
RRRIEGKVGERFNFHIPLILEDLICWNKYWNMTKANCIKNVPKNVSSSNWFQHFFITDDAELNKSAIQGLVIAQLKNDPQFKDQELNIEDIQADSTGHRLILGLAAKELISSNSLLSSLYKNIKYLMS
QKSVFVYNSTKQEIENLESPKDWYKELIKISYQDFEILEKYEKEENAELEEPYADTEPLKKKKKNINKNQESDDLKGQFIIPNKFPELKIQRNKLKLIVKDLLISKYAGTQKPKKFNNNLSAKDKILNSKS
KYYLLQDDKLIDKLVKLLEQGIISEALGSMDLDYQTTFRYNLCQGESLQVYEKDVVHIKALTKVQPKGLGDKIKSKLTWLKLDYEEIPAKENKKGFPDWLDLRVNQGIITIFGKPKRNNIGETKFQVKS
RTGYIIREFWILVQENQNQQVSQEKENVNPFSKIQKLALGFNLNKNNNKNQQIIEINSDSDLEVSKQDIQKSGEKEIQNKYQNQKSDKENHQDQIIENKQDGEGEVLSNEQKNSSDSGVPVIQK
KTSILRIKSQFKLNNNDDNEYQDIAKQFEEQNEDYDECKSENKSVISKSPDSHKNLIQSQDSDNKQLNKSQNSNKLIEEQIKKSDKNKIVKLNNKNKKGAYGQDQQESVYQKNLTSMFQFQKIDEI

SDLNDKKKILQEEDAQINQMLSELQKKKAAKQKQFIQKITVMGKILNLNKNKPYINNSCTNTEISQNSPSNLLLPQQNKQQTFKSQYYQLQHQKTSSESSPSNNNINNNDNNRKFQKFIIKKYIIP
YDTEYRLTTQRRYYRLRLEIAKNWRLIR*
>PPERSA_00121720
MLAQEFSDQGCQQFIITGSATGKVDKLNATHYCLVENFDLQKNCLTCRGPIKASSESMTHGNLYKMDKNTKVVIHVHNNKMWKRLLHKIPTTDKNAQYGTPEIAKEVERLFLETNVKDQKIFVL
EGHEDGIFSFGETFQEAYDIIYRIIINDEEI*
>PPERSA_00125930
MNVSLTLYGVRKVNHLIKNQWWAVYAHLIANRIRRLGVLAEIFLDDEDFVELQKLHNIKGIIISGGPNSTLDENSPIINKKIFDLQVPILGLCYGHQSICKTFGSKIVPGEVKEYGLATLNIDLNKNSPIF
KNLHNQQRIWMSHGDSVISVPEGFEIIGSTQSCEIAALQNLQKQIFSFQYHPEVTHTQNGVQMIKNFLFEICKCDDSQWDPKQYTQSIQNEIQQYIQNKKTGSKIFTLVSGGVDSTVAFTLLNKAL
GKDLNYGLFINNGLLRKGEFEFVDESIKKLGYDNFHSYDASEIFLNNLKEVYDPEEKRKIIGKTFIEVQRKVLQELNLNPQEWLLGQGTIYPDTIESKGTKHSDLIKTHHNRVEIIQDMINQGNVVEP
LQNLYKDEVRAVGLELGLPQNLIWRHPFPGPGLGVRCLCSEFDSVDQEYLQKFHHINKEITKALEEHDINGSILQVKSVGVQGDQRTYRHPACIYAQNIFQNFKQNYKEQKMGEQIKEDEEQNW
IWARLEHYSTQLTNKYDEINRVVILLKSRNYDAFAQNDNQNLKLQKVYITEKRISVLQMADHITMKHLIGNNLMKIVWQCPTVLIPLGQGKGESIVLRPVFSQEAMTAEFAKIDFKILEKLATEILD
QVQEITEVYYDITNKPPGTIEWE*
>PPERSA_00037920
MQQNNNNQQIQQKICDKCQFKSCLCEKIPQIELNAEFCLIYHQNEQFKATNTGQLLENAIKKVDKFLWSRIKPNPDLLLKLQSEKYQPYLLFPGNELHPSIQFPSENQEIVTEYKIEDKKQNEEQQE
QQQLQNEEQQLQQQQIDEIKNIKQFENQNLSQIKIPVKKCKIYIDKIPYFIIIDGVWKEANKIVKQSPYLQNIPRLELKPDKVSQFSLRRRQREDGLCTCEVGIYIIRNCLQEKEKAQQLEEYYEQFVQ
QFQKEQKKRYIRDEEYVIDKDTRQKILKSKFDNEVTQKKVKI*
>PPERSA_00036460
MDNLDTQLILQNLQHINKESNFMINNNIKINKVLKCGSIQMVMGHKLNINHFGAIYAGSIYSLAQQAGKFAIYSFQKEKVVVQIKKSEIQYLIPGKRDLYVEIKINNNEQQKIIQEVNEHGKSNITLE
FDIKEQNGTVVSKFKGHYIIEKKQNMPKL*
>PPERSA_00036150
MFQNNVIKNNFSSSIKLQSRETFEKLFKETQELSPFYHKDTLKYLSTHLDMNLIALLKLNATPQQLNNYYDQYLQERDIKKKAQINEAKDSQIVDKQTFLQQLNKYELYESYLYFLKDVEYKQNKKSL
NEIINEYIPILYPGIGSEAYHCVIKIAYGLDHDNQAEQILQGLAYLASAQALFPNEYGKSKDKGIFETLEEIHKQQEYFKKITHFDEPRVFNDKIIKLQIDDKFQDLVPNHFIIEGSFEEHIHKISLFLIKLFIE
TDNFLILHTVTGFYATKRLLKQLEKEEDKILVVDCFLKNVSAAYAIGDFIKIQNREKLEKRLQIPKNLSEWEEIINKANNSYDDHDNKLTYICKMEYNYLKENGYDENTYNLYRYAAAIQTKLLE*
>PPERSA_00036470
MINQQGDRVPFLTRNGVKIIDIKKGYCKVKLEYNDQILNESDQIDEGVIYSLAESISGIVFETYFDGKRYFPLVYRQSIEFLKIPPKVDLFVEQTFSQRQAEQIEEELNKKGKSKYKTTANVVDKEGNIY
AKSYGYYMGKSLNYKL*

>PPERSA_00042620
MGNQDQNSVIVSSSNKQSSLFYIYSYPVVFSPILLFSSEETGIAHNIIVSDDFTKLFVVSESDGLIIYDIQDIKNPVKKENLNFGDNHIYAAYYNSKSMAAIIEINTGIEIVSISSTYNYIFLGEVTYNNFQP
VSVAFSQNGDYLFVLDELLNQVLIFDISNLPSYSLVKSINLEYSPYQIEVRDKYAYLAAGYQGVIILDIQNVNNAQIIGQVQLPTLATFVTYIEEDDLLIASDLEYGLQIIQWDDIQYPVIINQADTLDKCY
QVVQEKNSQDFYVASGTAGIQGFRLKFSQTEQVIETLTENIPYHRIIKEINIQNLSYTYITQNAQYLFAVSTDGEFYSYNNSDKTSPVLSKQIAIGGIPKEIIVSISKNYAYVTLD*
>PPERSA_00125230
MSDTQNASSGLNVPRIEELLVQGGNYYTKQSFLEQEPGKYFQYSDFNFVIIATIIENIVKQRFDIYMNENVLEPLGISGSFNIDDLSQNQINNLACLYDIDDLGVLYESKNCFKKQKPDFTHLTNGYIV
GTNGGLFNPQGGLNLTLEELHQVLYMLYNDGISIKDKKQIIQKSSTDLMKTIQWQYDGNNAKQSSFIQAYGQGLQISTDKKKIDQIFPGRQVVGHTGQAYGLMSNMFFDAKNGNGYIFAHNG
MKQQPLFSNKSKFYKIEQDLIDLSYNALYTEKYQMSFLVAFRIIIGMGAYIGAIIFGVFFCYNKKQEKQKQQKKNDVLLNEELEKQEIK*
>PPERSA_00059730
MFAAIICFGTGLTTISLLAQTMARAEQLNEDIKQFVLVIPNLTGCFLRFYVGRGIAIEGGRKSALVCALLTFIGVLIMFIALISKDISTLQSSDEQFAFLVIGSFLSGSAIANFQLIVEMVMWYPLEIAGTV
QGIFAGVGNLFLGAFGLILINCLQNGLSLIGGYIIVLVLLVITLPLTYIYLQKTPLTQLIDQKMEENKATIIAEFCGQQNFPSKFHALKSMRDIPTIIICLNYFFCFGGFLSISSYLKGYIMAYHKTTVYEALF
QNFTFSCITSLTRVFTGYLTDKITGFLSTQISLIIIIISSLILSFSQSNSPHSFMHIGGLLFLAIGIGFMMAGNYKWIVEIRYNHILAIGMLAGFFGALGGPILQLTLFDESLAKGGQYLPLAFLFFTTLGIVILI
INQVLHIDQIKKQKASMYK*
>PPERSA_00010290
MEDLRKNIEELNLKQVKTYIQTGNIVFKSDKNETQLKELIQNQIKQKYNFEVTTIVIKQVDLVQILQKNPFLQIEKSKDQQQNKNTEEEIISIQENEINIQNTKKQQKVKTVKNNNNINKKTDYLVFFE
KKIDDEILQNQKLNLDKIQDKFEINPIFTNILYLKVYDGYGATKLNINFFEKQLKIKGTVRNLNTCQKVIEMLNQIK*
>PPERSA_00117680
MDIKMNSEQINLSEIEKYQFQIFKPASLLVIVTIFVWLAMNATRFPIISKLKDLQVLNYPTQKEKILQSVSGPSNNFINLFEFPVLFYLACVVIAVCKINSGSFMSLAYGFVFFRAIHSLIQSTYNLIMHR
FMVYYVISCLILFIMWGNILYHIW*
>PPERSA_00050710
MLQKNEQITQKAHYIESLQPGYSIKPGQVDQFYTEAYEQQIQEISDLQIKNQTVKIGIDFGNVIVGSDPNANDPDEDTMFGENYLQVKAIPDAIEGVKFLVEKFGADNIYIVSKAKQKMQGKSLN
WMQHYDFYNKTGILEKHMIKWTSQIKWMNLQ*
>PPERSA_00009640
MNTYTQILKAQIDTDQKEFDNGISRNNQILIQTNQSIQNQESSEVIIDEIEEFRQQDFNQNGIQLLKEQSSKYQQVTKKIEKYNKKGRRNSNKSVNIQSSQKTIPANGNQNNTDLKEQQQIKQPN
YKQNKNNKNSKQINKNQCQTNYPQNTKRFLRSHSKQLLKLKTQISETPRTKKIHDQVLQMKLNLLTSYQLLQKKIAQKKLWRPEILSEILDFAFQNTTTHLKKALHAQEEKLRSSLFLMNKQKQNQ
LLTWNYLLVDKSKKVQFQTDLLSQNIKSLENYGKILKKGVGRNPPKEVIQNIKKEFMMFEYEPGFFIKKQGMEFYDKENYTQAIEQFNVYLEQYNHTNKTAIINRGLSYMKLKKFDKALEDLQNCH

KLYPQCHTGFMYSGWVKYQQSNLESALYDLLIAQELNNQDIYVYIYLSLTFMKLKVFDKSEEACLNALKLNPNNVFAQAVLHKIKTQKVFAKNQQKQLNNNIKIMNNIQEQNQILQNYRIKLQQE
IDQQLEQQIIPQIEE*
>PPERSA_00076020
MKQIFTRLKSHNYLKYQKSIQNISEKYQVECFQKLNPPKVPGNYLGIINKGIENLYKENSLLAVSPSVYDIENQDEKLSVISCLQSFEKMYHVTVLSNFSIGFDKYSMTYSKENLEKSTFKNQFYLLTED
ELEIGYQKSDKLLKKLNLKDNQRIILCTTSDYIQKSKNKLQKNQNTGLGYYIDDNKIKISELYVVDDNGKMKPISVEDAYALSMKINQKKLYSYQDLVPRTISILPVAQGCQAKCPFCFSHTSVSKDVK
QKILNKHIIEATLQYAKQRGAERAVITGGGEPTMLPFHYIIKLIKQCKKHFDRVIMITNGYNLGHIEQEQERKAALQALDEAGLNILAVSRHDIKTKKLMNLETKSHKIARSLQGMQFLRNLQLRWV
CVIQKGGVQDEESLVRYLDDVVETGGCDQINFKELYVASSQESVYYEEEGNEFCRKNQVSLSLITEFLDKNNAKVLKAREFVGLGI*
